# Group-specific cellular metabolism in Medulloblastoma

**DOI:** 10.1186/s12967-023-04211-6

**Published:** 2023-06-05

**Authors:** Viktoria L. E. Funke, Carolin Walter, Viktoria Melcher, Lanying Wei, Sarah Sandmann, Marc Hotfilder, Julian Varghese, Natalie Jäger, Marcel Kool, David T. W. Jones, Stefan M. Pfister, Till Milde, Martin Mynarek, Stefan Rutkowski, Jochen Seggewiss, Daniela Jeising, Flavia W. de Faria, Thorsten Marquardt, Thomas K. Albert, Ulrich Schüller, Kornelius Kerl

**Affiliations:** 1 Department of Pediatric Hematology and Oncology, University Children’s Hospital Münster, Albert-Schweitzer-Campus 1, 48149 Münster, Germany; 2 Institute of Medical Informatics, University of Münster, 48149 Münster, Germany; 3 Hopp Children’s Cancer Center Heidelberg (KiTZ), Heidelberg, Germany; 4 Division of Pediatric Neurooncology, German Cancer Research Center (DKFZ), German Cancer Consortium (DKTK), Heidelberg, Germany; 5 Princess Máxima Center for Pediatric Oncology, Utrecht, The Netherlands; 6 Division of Pediatric Glioma Research, German Cancer Research Center (DKFZ), Heidelberg, Germany; 7 Department of Pediatric Oncology, Hematology and Immunology, Heidelberg University Hospital, Heidelberg, Germany; 8 Clinical Cooperation Unit Pediatric Oncology, German Cancer Research Center (DKFZ) and German Consortium for Translational Cancer Research (DKTK), Heidelberg, Germany; 9 Department of Pediatric Hematology and Oncology, University Medical Center Hamburg-Eppendorf, 20251 Hamburg, Germany; 10 Mildred Scheel Cancer Career Center HaTriCS4, University Medical Center Hamburg-Eppendorf, Hamburg, Germany; 11 Institute of Human Genetics, University Hospital Münster, Münster, Germany; 12 Department of General Pediatrics, Metabolic Diseases, University Children’s Hospital Münster, 48149 Münster, Germany; 13 Research Institute Children’s Cancer Center, 20251 Hamburg, Germany; 14 Institute of Neuropathology, University Medical Center Hamburg-Eppendorf, 20251 Hamburg, Germany

**Keywords:** Medulloblastoma, Metabolism, Inositol phosphates, Nucleotides, RNA-Seq

## Abstract

**Background:**

Cancer metabolism influences multiple aspects of tumorigenesis and causes diversity across malignancies. Although comprehensive research has extended our knowledge of molecular subgroups in medulloblastoma (MB), discrete analysis of metabolic heterogeneity is currently lacking. This study seeks to improve our understanding of metabolic phenotypes in MB and their impact on patients’ outcomes.

**Methods:**

Data from four independent MB cohorts encompassing 1,288 patients were analysed. We explored metabolic characteristics of 902 patients (ICGC and MAGIC cohorts) on bulk RNA level. Moreover, data from 491 patients (ICGC cohort) were searched for DNA alterations in genes regulating cell metabolism. To determine the role of intratumoral metabolic differences, we examined single-cell RNA-sequencing (scRNA-seq) data from 34 additional patients. Findings on metabolic heterogeneity were correlated to clinical data.

**Results:**

Established MB groups exhibit substantial differences in metabolic gene expression. By employing unsupervised analyses, we identified three clusters of group 3 and 4 samples with distinct metabolic features in ICGC and MAGIC cohorts. Analysis of scRNA-seq data confirmed our results of intertumoral heterogeneity underlying the according differences in metabolic gene expression. On DNA level, we discovered clear associations between altered regulatory genes involved in MB development and lipid metabolism. Additionally, we determined the prognostic value of metabolic gene expression in MB and showed that expression of genes involved in metabolism of inositol phosphates and nucleotides correlates with patient survival.

**Conclusion:**

Our research underlines the biological and clinical relevance of metabolic alterations in MB. Thus, distinct metabolic signatures presented here might be the first step towards future metabolism-targeted therapeutic options.

**Graphical Abstract:**

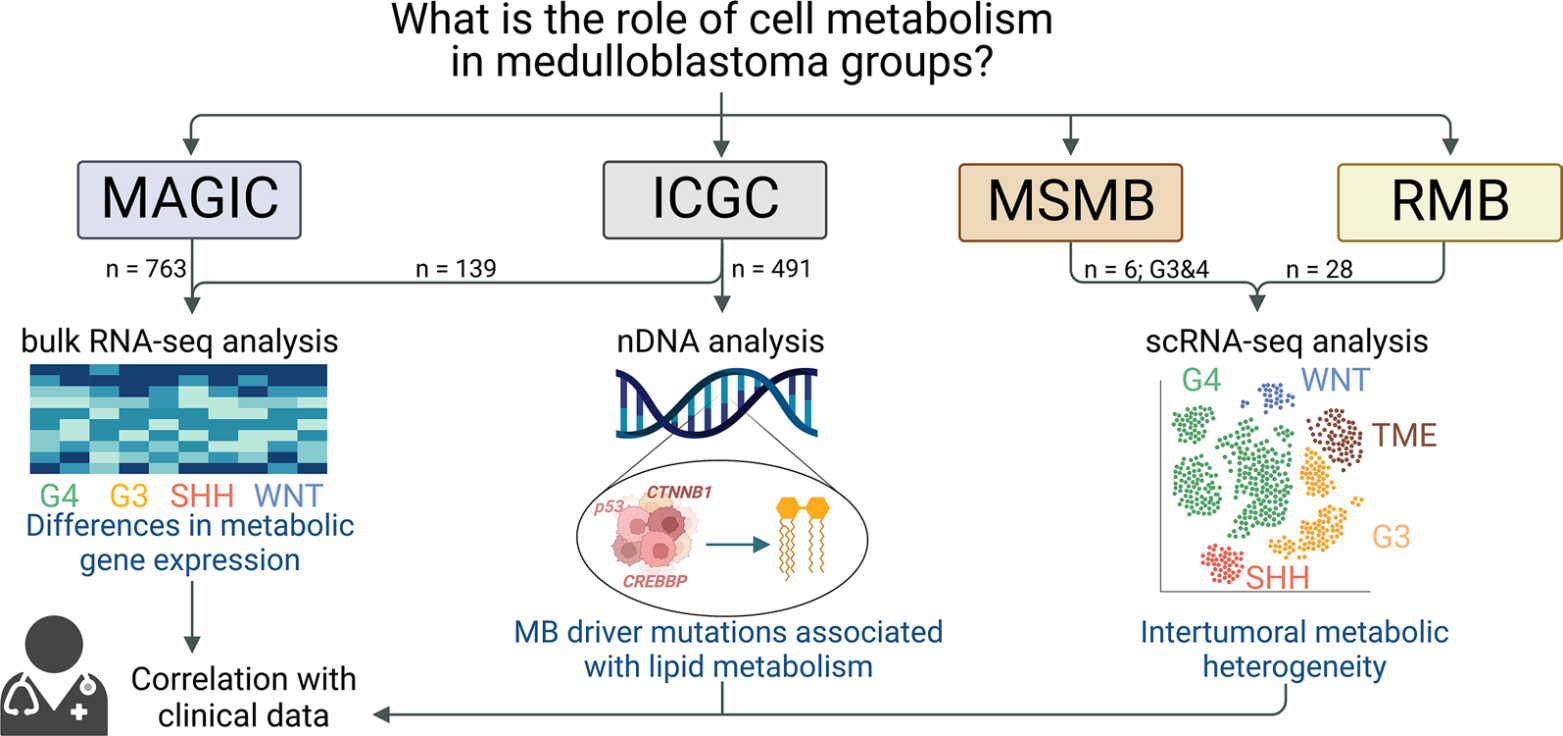

**Supplementary Information:**

The online version contains supplementary material available at 10.1186/s12967-023-04211-6.

## Background

Medulloblastoma (MB) is one of the most common malignant paediatric brain tumour types and a highly heterogeneous tumour entity. In addition to the four consensus molecular groups, WNT, SHH, group 3 (G3), and group 4 (G4), extensive analyses of multiomics data have resulted in an even further subdivision into numerous subgroups [1–4]. Overactivation of WNT/β-catenin and SHH signalling pathways characterise WNT and SHH MB, respectively, and are associated with a good (WNT), intermediate (SHH, *TP53*-wildtype) and poor (SHH, *TP53*-mutated) prognosis [5–7]. Meanwhile, G3/G4 MB correlate with an intermediate (G4) and poor (G3) patient outcome [5], share some overlapping features and are not consistently separable [2, 6]. Recently, Sharma et al. [2] introduced eight distinct G3/G4 consensus subgroups based on the analysis of DNA methylation data. Of these, subgroups II, III, and V were identified as high-risk MB exhibiting a particularly unfavourable prognosis and frequently showing amplification of either *MYC* or *MYCN*.

While molecular risk stratification has significantly improved our understanding of MB, it is not yet fully elucidated what drives heterogeneity and gradients, especially among G3/G4 MB [2, 6]. One aspect contributing to the diversity among malignancies, in general, is aberrant cell metabolism [8]. Various factors shape the metabolic phenotype, including cell-intrinsic influences (e.g. the cell of origin, oncogenome or deregulated signalling and metabolic pathways) and extracellular factors such as the tumour microenvironment (TME) or nutrient availability [9–12]. It has been established that MB emphasises anabolic pathways similar to progenitor cells in the developing cerebellum to promote tumour growth and ensure tumour survival [13, 14]. However, a few studies imply that metabolic patterns might not be consistent across MB groups [15, 16]. This is also exemplified in our previous work showing extensive metabolic reprogramming in the tumour of a mouse model for SHH MB, which affected lipid metabolism, nucleotide metabolism, and oxidative phosphorylation (OXPHOS) [17]. To answer whether specific alterations in metabolic dependencies could potentially serve as future therapeutic targets [18], detailed knowledge about the extent of metabolic diversity in MB and underlying molecular mechanisms is needed. Therefore, this study comprehensively explored metabolic differences between and within MB groups on a genomic and transcriptomic level. Focusing on G3/G4 MB revealed three metabolic clusters that significantly impact patients’ survival. Lastly, upregulation of genes involved in the metabolism of nucleotides and inositol phosphate (IP) compounds correlated with patients’ outcomes.

## Methods

### Patient samples

Data from 1288 MB patients derived from four independent cohorts were included in this study. ICGC cohort encompassed whole-genome sequencing (WGS) data of 491 patients. Normalised RNA expression values of 139 ICGC tumour samples retrieved from the European Genome-phenome Archive were explored in bulk RNA analysis. Supplements from Northcott et al. listing nDNA mutations in ICGC cohort was searched for alterations in all 491 patients [19]. Microarray data from 763 tumour samples from MAGIC cohort were obtained from Gene Expression Omnibus GEO [20]. Clinical data and group annotation were added from a previous publication [4]. Both cohorts comprised all four MB groups. Metabolic heterogeneity on single-cell RNA level was explored using six additional human MB samples (two G3, four G4) from patients treated in Münster, Germany, with ethical committee agreement (2017-261-f-S, Münster, Germany). This dataset is referred to as MSMB in the graphical abstract and the main text. Findings were validated by exploring a second single-cell RNA cohort encompassing data from 28 patients published by Riemondy et al., abbreviated as RMB dataset [21].

### Single-cell RNA sequencing

Six human MB samples were dissociated and further processed into single-cell suspensions of vital tumour cells as described [17]. We applied chromium technology (10× Genomics) for single-cell capture, barcoding and cDNA amplification. The Library Bead Kit and i7 Multiplex Kit were employed for library generation. Quality controls were performed with a Tapestation 2000 instrument (Agilent Technologies). Sequencing of samples was conducted at Core Facility Genomics, University Hospital Münster, on an Illumina NextSeq 500 instrument utilising High Output Kit v2 with 75 cycles. Single-cell RNA sequencing (scRNA-seq) data are available on Gene Expression Omnibus GEO [22].

### Data analysis

The bioinformatic data analyses were conducted using R-versions 4.0.5 and 4.1.3 [23, 24]. A list of metabolic genes compiled from KEGG and REACTOME metabolic pathways was obtained from the cancer cell metabolism gene database (ccmGDB) [25]. Gene signatures of metabolic pathways previously published by Rosario et al. [26] were used to validate bulk RNA and nDNA analyses and for survival analyses. To explore bulk and single-cell RNA data, canonical pathway analyses of differentially expressed genes (DEGs) were performed using QIAGEN IPA (QIAGEN Inc., https://digitalinsights.qiagen.com/IPA) [27]. Bulk RNA analysis and selected findings from single-cell RNA analysis were validated using Metascape [28]. The graphical abstract was created using BioRender.com. Further details on analyses and packages used are provided in the Additional file 1.

## Results

### Metabolic gene expression in MB subgroups

**Fig. 1:**
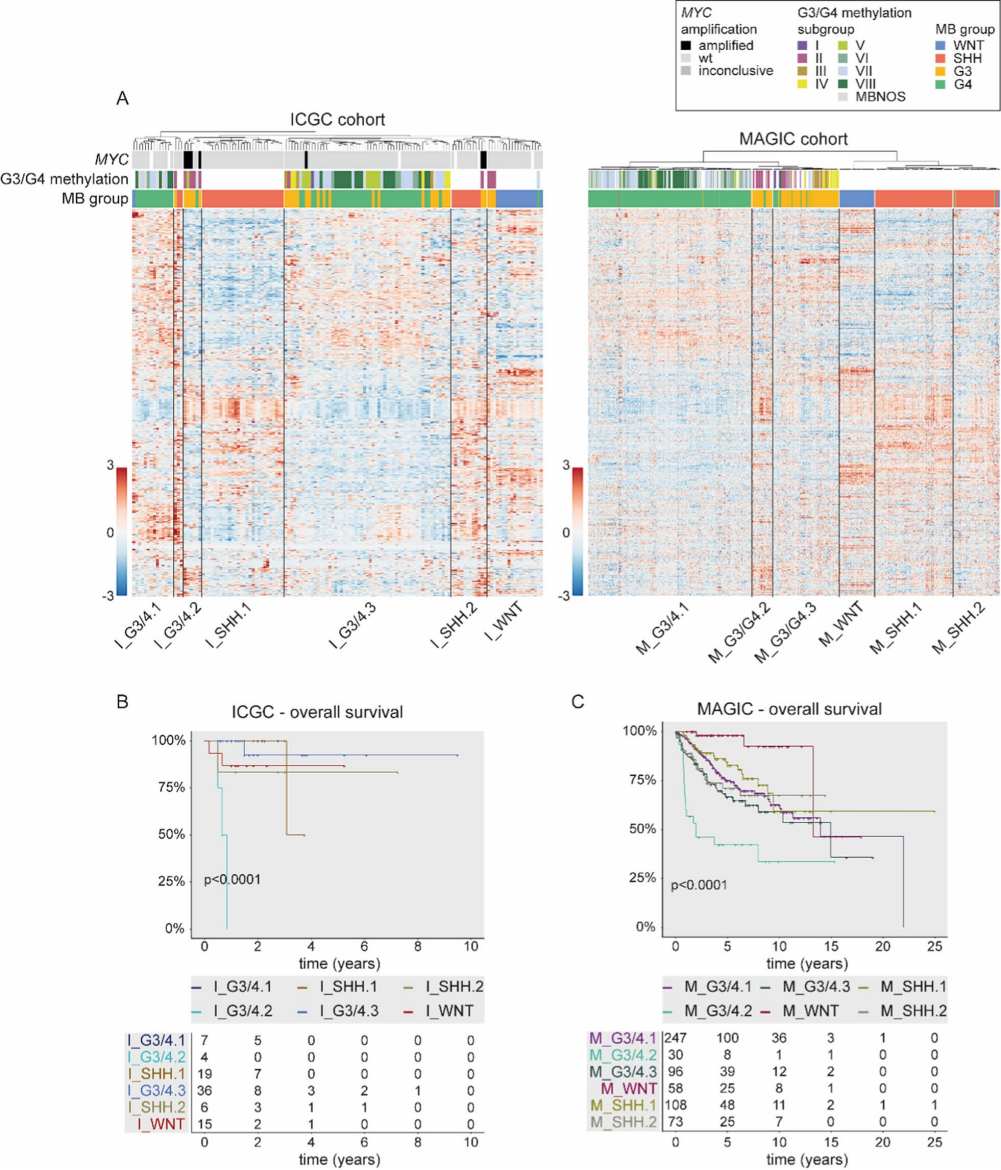
Established MB groups differ in metabolic gene expression with association to patients’ prognoses. **A** Heatmaps showing the expression of metabolic genes from the ccmGDB across 139 samples from the ICGC cohort and 763 samples from the MAGIC cohort ordered by unsupervised hierarchical clustering. For the MAGIC cohort, genes are in the same order as for the ICGC cohort. Annotation bars on the column side illustrate the amplification status of MYC (only ICGC), correlation with published methylation G3/G4 subgroups [2], and the samples’ MB group affiliation from top to bottom. **B, C** Overall survival of patients from **B** ICGC and **C** MAGIC cohort. Patients have been grouped into metabolic clusters identified in **A**. Log-rank test was used to calculate *p*-values, and *p* < 0.05 was considered significant. MBNOS = MB not other specified; wt = wildtype

In order to study characteristics of cell metabolism in MB, we evaluated RNA expression levels of 2071 genes from ccmGDB associated with cell metabolism (called “metabolic genes” thereafter) across 139 MB samples from the ICGC cohort [25]. Unsupervised hierarchical clustering was performed, and silhouette method revealed an optimal number of three clusters for the resulting heatmap (Additional file 1: Fig. S1B). However, as a separation into three clusters would have implicated a strong mixing of the according consensus MB groups, the cohort was divided into seven metabolic clusters instead, including one WNT, two SHH and three G3/G4 clusters (Fig. 1A; Additional file 1: Fig. S1D). One cluster was excluded as potential outlier, as described in the Additional file 1. Patients from cluster I_G3/4.2 (predominantly G3) had a significantly shorter progression-free and overall survival (Fig. 1B; Additional file 3: Table S2).

To validate this metabolic classification system, we used the 763-sample MAGIC cohort. Here, we were able to identify six metabolic clusters that were associated with different survival of affected patients (Fig. 1A, C). Kaplan–Meier survival curves highlighted M_G3/4.2, which is dominated by G3 MB (G3 n = 33; G4 n = 4), as a “high-risk metabolic cluster” with significantly shorter overall survival, comparable with I_G3/4.2 (Fig. 1C; Additional file 1: Fig. S3A). To further molecularly classify these two “metabolic high-risk clusters”, we compared our clustering with published G3/G4 methylation subgroups from Sharma et al. [2] and found that I_G3/4.2 (n = 6) and M_G3/4.2 (n = 37) comprised mainly samples from consensus methylation subgroup II (I_G3/4.2 n = 4; M_G3/4.2 n = 21). Accordingly, five I_G3/4.2 samples showed amplification of either *MYC* or *MYCN* and 30 M_G3/4.2 samples had been categorised as G3γ earlier [4]. I_G3/4.1, I_G3/4.3, and M_G3/4.1 encompassed several samples belonging to methylation subgroup VII or VIII [2]. For M_G3/4.3, a similar trend was not as clear. Compared to M_G3/4.1 and M_G3/4.2, this cluster also included larger fractions of methylation subgroups III and IV, which most likely resembles samples included in I_G3/4.3 in the other cohort (Fig. 1A; Additional file 1: Fig. S1).

In order to rule out that this clustering is reliant on the gene list from ccmGDB, we performed a second analysis using a collection of 1771 metabolic genes based on the work of Rosario et al. [26]. While ccmGDB also considers genes involved in RNA metabolism, Rosario et al. comprised a complete set of genes belonging to certain pathways, for example, OXPHOS, but also lipid and carbohydrate metabolic processes (Additional file 1: Fig. S2A–C). Despite these differences in essential metabolic categories, we received a similar clustering in both cohorts using the second gene list (Additional file 1: Fig. S2D, E). This finding supports the idea that the metabolic clusters depicted in Fig. 1 are not solely dependent on one specific set of metabolic genes.

Next, we aimed to get more detailed insights into functional networks underlying the described clusters of G3/G4 MB. Therefore, we compared the transcriptome of all clusters and only G3/G4 clusters performing IPA canonical pathway analyses and using Metascape to gain a comprehensive understanding of enriched genes and pathways.

**Fig. 2:**
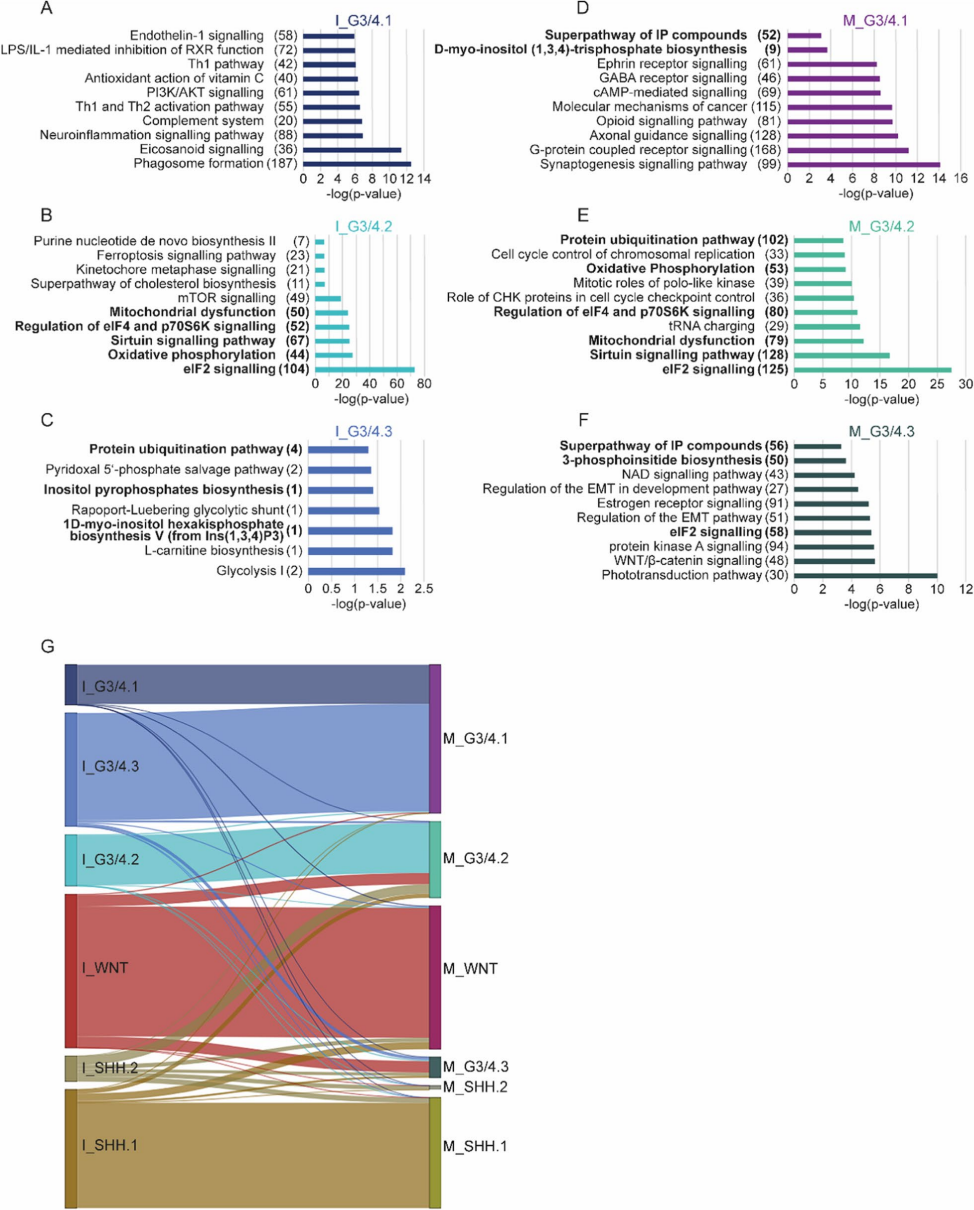
Functional characterisation of metabolic G3/G4 clusters in examined cohorts. IPA canonical pathway analysis of upregulated DEGs comparing solely clusters dominated by G3/G4 MB has been performed. Pathways are presented for **A** I_G3/4.1, **B** I_G3/4.2, **C** I_G3/4.3, **D** M_G3/4.1, **E** M_G3/4.2, **F** M_G3/4.3. Pathways that overlap in content across clusters are printed in bold. Behind each pathway, the number of DEGs matching this pathway is listed in brackets. **G** Sankey plot illustrating relationships between metabolic clusters in ICGC and MAGIC based on DEGs of each cluster as outlined in the Additional file 1. The width of each bar representing a cluster refers to the total number of DEGs which overlap with clusters from the other cohort. IP = inositol phosphate; EMT = epithelial–mesenchymal transition

The upregulated DEGs of I_G3/4.1 indicated enrichment of genes associated with inflammation and immune response (Fig. 2A; Additional file 1). This result is particularly interesting as immune and stromal cells are known to strongly impact nutrient availability and cancer cell metabolism [12, 29]. Computational quantification of cell types from the TME employing MCP-counter [30] revealed significant differences in the abundance of various cell populations across metabolic clusters (Additional file 1: Fig. S6; Additional file 6: Table S5). Especially the amount of B and T cells, cells from the monocytic lineage and neutrophils varied among clusters from both cohorts when contrasting all and only G3/G4 clusters. As depicted in Additional file 1: Fig. S6, I_G3/4.1 exhibited comparably high abundance scores for these cell types.

In contrast, IPA canonical pathways such as OXPHOS, sirtuin signalling pathway, eIF2 signalling and purine biosynthesis implied a relevant role of energy, mRNA and nucleotide metabolism in I_G3/4.2 (Fig. 2B). I_G3/4.3 exhibited 128 upregulated DEGs, which are involved in glycolysis and the metabolism of IP (Fig. 2C; Additional file 5: Table S4). Although OXPHOS and IP metabolism were less clearly recognisable in the results from Metascape, we again found pathways highlighting RNA and nucleotide metabolism in I_G3/4.2 (Additional file 1: Figs. S4A, S5A, B). Analysis of upregulated DEGs of I_G3/4.3 when comparing only G3/G4 clusters resulted in several pathways pointing at energy deficiency, such as protein modification and catabolism or mitophagy (Additional file 1: Fig. S5C) [31]. However, this latter result should be interpreted cautiously due to the small number of DEGs and lower gene expression values used for this cluster (Additional file 1).

To compare our findings to the clustering from the MAGIC cohort, we repeated all analytical steps with DEG lists derived from this cohort. Additionally, we constructed a Sankey plot using each cluster’s top 250 unique upregulated DEGs (Fig. 2G; Additional file 1). DEG analyses indicated a resemblance between I_G3/4.2 and M_G3/4.2 when employing both QIAGEN IPA and Metascape (Fig. 2B, E; Additional file 1: Fig. S4). As presented in the Sankey plot, further similarities between I_G3/4.1, I_G3/4.3 and M_G3/4.1, as well as the SHH clusters I_SHH.1 and M_SHH.1 and the WNT clusters, could be observed (Fig. 2G). The similarity between the WNT clusters M_WNT and I_WNT is also evident when comparing gene expression patterns in bulk RNA heatmaps (Fig. 1A), indicating a distinct metabolic gene expression profile. I_SHH.2, M_G3/4.3 and M_SHH.2 appeared to have only few unique DEGs overlapping with other clusters. One possible explanation for this observation might be variable data processing before our study or differences in cohort size resulting in a more homogenous clustering of established MB groups in the MAGIC cohort. The latter argument is also supported by canonical pathways of M_G3/4.1 and M_G3/4.3 when only comparing G3/G4 clusters (Fig. 2D, F). While top metabolic pathways in these clusters refer to IP metabolism, top canonical pathways, in general, resemble characteristic biological processes enriched in G4 MB (M_G3/4.1) and G3α and β (M_G3/4.3) [4]. As described above, G3/G4 samples of methylation subgroup III/IV (mostly equatable with G3α and β) clustered together with samples from subgroups VII and VIII (mostly G4) in I_G3/4.3 while forming a separate cluster in M_G3/4.3 [6]. Because two-thirds of I_G3/4.3 comprises samples classified as G4 MB, a resemblance of I_G3/4.3 and M_G3/4.3 might be concealed when only looking at unique DEGs. Creating a plot similar to Fig. 2G without focusing on unique genes provides evidence of overlaps between I_G3/4.3 and M_G3/4.3 (Additional file 1: Fig. S3C).

This idea seems to hold concerning the SHH clusters, too. Considering upregulated DEGs, in general, demonstrates that metabolic gene expression programs of identified SHH clusters are not as easily distinguishable. Meanwhile, Fig. 2G implies a resemblance between I_SHH.1 and M_SHH.1. M_SHH.1 consists mainly of samples from SHHα and SHHδ, while large parts of M_SHH.2 are made up of SHHβ and SHHγ. Interestingly, this suggests a division of clusters by age since SHHβ and SHHγ primarily occur in infants, while SHHα and SHHδ affect mostly older children and adults [4]. Data regarding these subgroups were not available for ICGC cohort. However, patients from I_SHH.1 were significantly older compared to all other ICGC clusters, with a mean age of 22.6 years, while the mean age of patients from I_SHH.2 was 14.6 years. Even though pairwise Wilcoxon test comparing only I_SHH.1 and I_SHH.2 was not significant, this indicates that samples from I_SHH.1 likely classify as SHHδ while I_SHH.2 contains a combination of samples from different age groups (Additional file 1: Fig. S1; Additional file 3: Table S2).

Altogether, our analyses reveal apparent differences in metabolic gene expression between the established MB groups. Moreover, we discovered a separation of G3/G4 MB into three clusters displaying distinct metabolic programs. Enhanced metabolism of RNA and nucleotides together with OXPHOS distinguished high-risk MB patients from others.

### Metabolic programs of G3/G4 MB reflect intertumoral metabolic heterogeneity

**Fig. 3:**
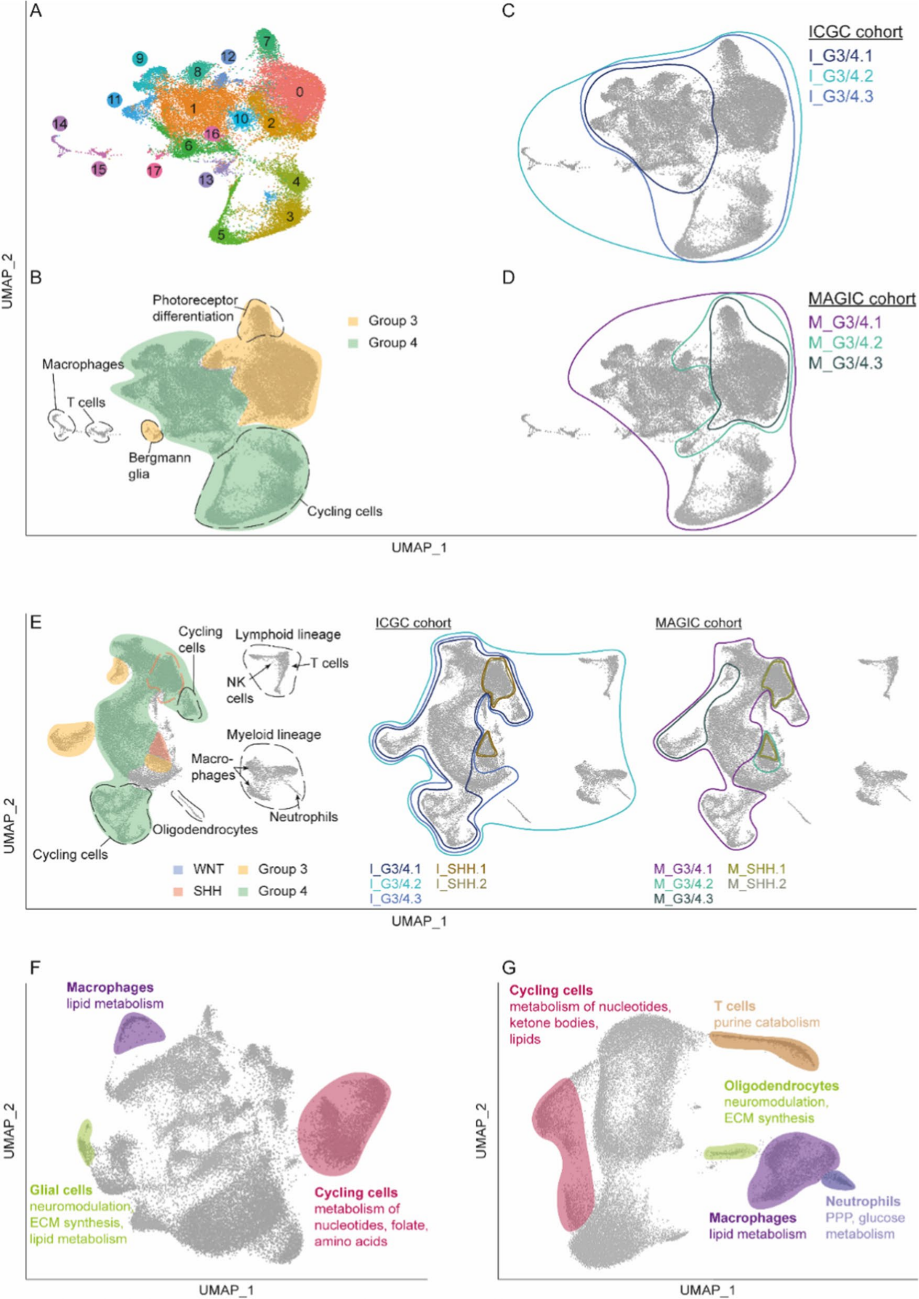
Intertumoral metabolic heterogeneity in G3/G4 MB. **A** Integrated clustering of six (two G3, four G4) human MB single-cell transcriptomes is shown in a two-dimensional UMAP plot. **B** Cell clusters have been annotated regarding their affiliation to G3 and G4 MB or as non-malignant TME cells using cell counts and different marker gene signatures (Additional file 1: Figs. S7, S8). **C**, **D** UMAP plots showing overlaps between G3/G4 MB groups and the metabolic gene signatures obtained from ICGC or MAGIC. **E** The analysis has been repeated using a second scRNA-seq dataset derived from Riemondy et al., encompassing samples from all four MB groups. Moreover, UMAPs based on single-cell RNA expression of metabolic genes from ccmGDB, also used for bulk RNA analysis, are shown for **F** MSMB and **G** RMB cohorts. Annotation of cell types from the TME and metabolic processes highlighted in these metabolic UMAPs has been performed as described in the Additional file 1. ECM = extracellular matrix; PPP = pentose phosphate pathway

We performed scRNA-seq on six human G3/G4 MB samples (n = 2 in G3; n = 4 in G4) to explore metabolic clusters at single-cell level. This analysis revealed 18 distinct cell clusters, most of which were malignant cells, which in turn separated according to groups G3 or G4 (Fig. 3A, B; Additional file 1: Figs. S7–8).

Using gene signatures for each of the metabolic G3/G4 clusters from bulk RNA analysis (see Additional file 1), we next addressed the question of whether these clusters represent inter- or intratumoral differences. Signatures showed few overlaps (Additional file 1: Fig. S9A), confirming their suitability to represent the individual metabolic clusters. Expression patterns of examined gene signatures did not highlight different cell types reflecting distinct metabolic phenotypes within one group. Instead, a resemblance to the expression of established marker genes for G3/G4 MB was found. In line with the results from bulk RNA analysis, one exception was most likely I_G3/4.1, whose gene expression pattern highlighted cells from the TME in a direct comparison of G3/G4 MB clusters. Signatures of metabolic clusters consisting mainly of WNT or SHH MB samples exhibited only low gene expression levels (Fig. 3C, D; Additional file 1: Fig. S9).

Subsequently, we aimed to validate our findings utilising a published scRNA-seq cohort comprising data from 28 MB patients. We identified 19 clusters which were annotated regarding MB group and cell type affiliation. Despite a greater overlap of MB groups compared to the smaller cohort, differences in gene expression patterns were still detectable for SHH, G3 and G4 MB. WNT MB was represented with only one sample explaining why a unique gene expression pattern was absent. As can be seen from Fig. 3E, the similarity of the metabolic signatures’ gene expression and MB group markers was reproducible in this cohort. Strikingly, cluster 10 displayed strong expression of genes characterising M_G3/4.3, while expression of DEGs from M_G3/4.2 was instead observed in cells from cluster 4. When looking at the number of cells from different samples per cluster (Additional file 7: Table S6), it is noticeable that most cells in cluster 10 are derived from one sample classified as G3α [21]. Therefore, one can conclude that the differences in metabolic gene expression between different subgroups of G3 MB described for the bulk RNA clustering are also detectable in our scRNA-seq data.

Intending to address the possibility of intratumoral metabolic heterogeneity, we created new UMAPs based solely on metabolic genes (Additional file 1: Figs. S10A, S11A). Here, we observed TME and cycling cells displaying discrete metabolic features distinguishing them from other cells (Fig. 3F, G). Unique genes (e.g. *TK1*, *DHFR*, *DUT*) and the corresponding canonical pathways indicated the importance of the metabolism of nucleotides, folate and amino acids in cycling cells [32]. Pathways pointing at nucleotide metabolism were also present in the according clusters in RMB dataset (clusters 7, 8), accompanied by lipid metabolic processes (clusters 8, 10) and upregulated DEGs relevant for the metabolism of ketone bodies (cluster 10). In clusters of glial cells, unique genes (MSMB: *LCAT*, *PLTP*; both datasets: *BCAN*, *PTGDS*) were involved in neuromodulation, extracellular matrix synthesis, and lipid metabolism [33–36]. The top canonical pathways of macrophage clusters’ unique DEGs implied a relevant role of various lipid metabolic processes (e.g. phospholipases, phosphatidylglycerol biosynthesis, sphingosine and sphingosine-1-phosphate metabolism). This became even more apparent when analysing the according genes using Metascape. Here, the metabolism of lipids was identified as the top pathway in both datasets. T cells did not form a separate cluster in the metabolic UMAP of MSMB cohort but in the larger RMB cohort. In the latter, they exhibited only one upregulated unique gene involved in purine catabolism [32]. Earlier studies on the metabolism of neutrophils mainly reported a glycolytic phenotype. Nonetheless, it has been proposed that immature neutrophils can rely on OXPHOS to sustain the production of reactive oxygen species when glucose metabolism via the pentose phosphate pathway (PPP) is restricted [37, 38]. In our analysis of the RMB dataset, canonical pathways of upregulated DEGs in metabolic cluster 13 encompassed the PPP and pathways pointing at glucose metabolism, while oxidative phosphorylation was not among the significant pathways of this cluster (Fig. 3F, G; Additional file 7: Table S6).

When it comes to tumour cell clusters, the metabolic UMAP of the MSMB dataset depicted inter-sample differences in cells’ clustering, suggesting patient-specific metabolic features (Additional file 1: Fig. S10B; Additional file 7: Table S6). G3 MB clusters exhibited upregulated DEGs involved in the metabolism of nucleotides and carbohydrates, while G4 clusters expressed many genes associated with the metabolism of lipids and secondary metabolites, such as IP compounds. However, a similar separation of samples and MB groups was not visible in the second cohort from Riemondy et al. (Additional file 1: Fig. S11; Additional file 7: Table S6).

These results suggest that metabolic characteristics previously identified are also present in scRNA-seq samples and that intertumoral instead of intratumoral heterogeneity of metabolic patterns determines our previous findings.

### DNA aberrations associated with cell metabolism in MB

**Fig. 4:**
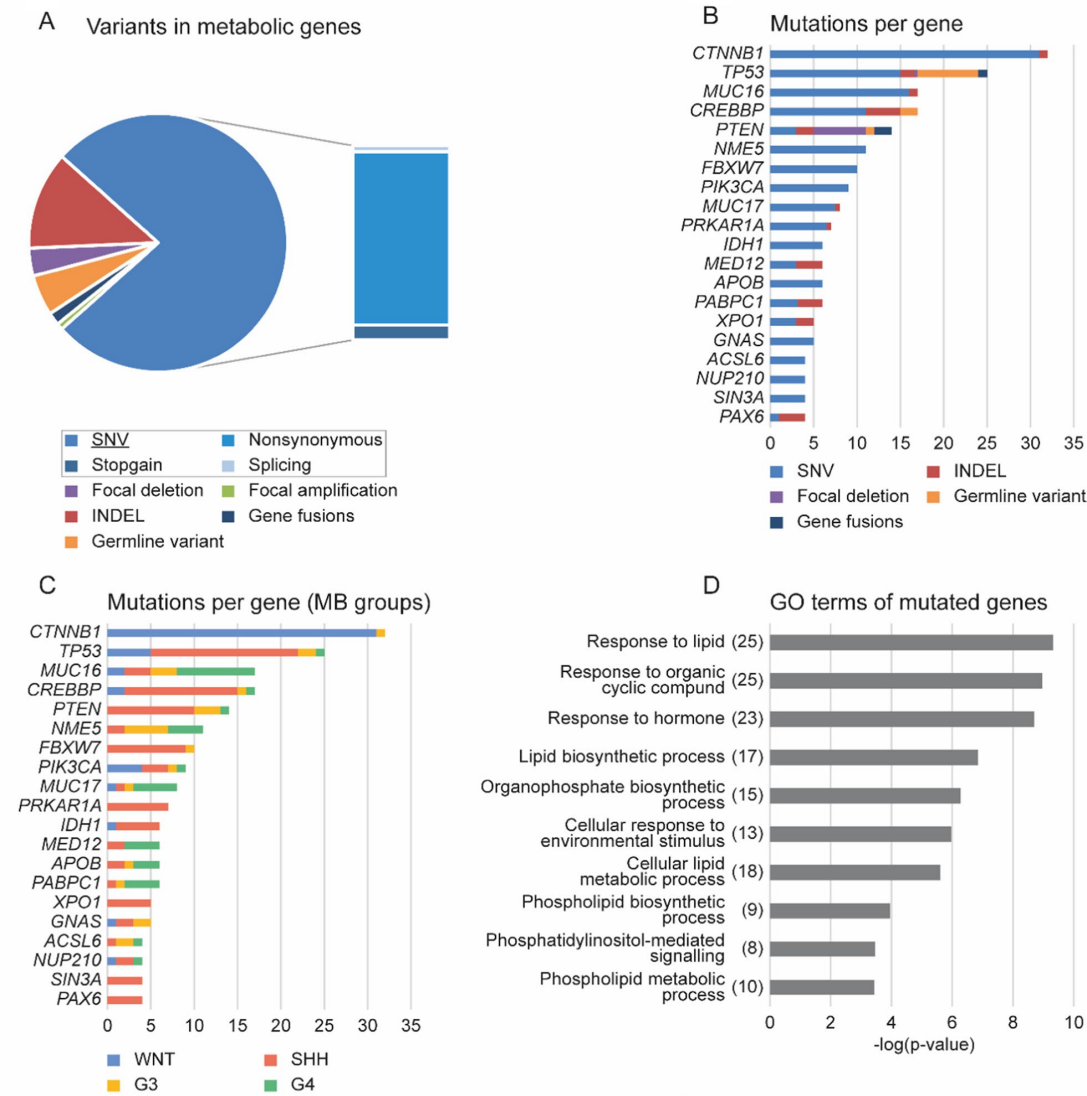
Mutation spectrum of metabolic genes in MB. Metabolic genes from ccmGDB were analysed for nDNA aberrations, and 374 variants in 62 genes were detected. **A** Mutation type of all variants detected is shown. SNVs have been further classified as nonsynonymous, splicing and stopgain mutations. **B** and **C** show the top 20 mutated genes, including the number of samples in which every depicted gene was mutated. **B** refers to the mutation type, and **C** to the MB group affiliation of the mutated samples. **D** GO analysis of all mutated genes using ToppGene Suite (https://toppgene.cchmc.org/). The number of DEGs found for each GO term is shown in brackets. SNV = single nucleotide variant; INDEL = insertions and deletions

Mutations in various genes can drive altered tumour metabolism and contribute to different metabolic phenotypes. Therefore, we searched 2462 previously published nuclear DNA (nDNA) mutations [19] detected in all 491 patients of the ICGC cohort for mutations in metabolic genes from ccmGDB. 374 mutations in 62 metabolic genes were detected. Single nucleotide variants (SNVs) comprised the largest fraction of detected variants. Of these, 256 SNVs had been classified as nonsynonymous variants, nine as splicing variants and 22 as stopgain mutations (Fig. 4A). The top 20 highly aberrant genes are shown in Fig. 4B, C. Many of these genes (10 of the top 20) appear to be regulatory genes relevant to carcinogenesis and MB development. This is evident in the enrichment of mutations in *CTNNB1* in WNT MB and *TP53* or *CREBBP* in mainly SHH samples (Fig. 4C) [19]. Gene Ontology (GO) analysis of all mutated metabolic genes revealed a strong association with lipid metabolism and aberrations in genes involved in IP metabolism (Fig. 4D).

In line with our bulk RNA analysis, we also searched for nDNA variants based on the second list of metabolic genes [26] (Additional file 1: Fig. S12). Here, we observed variants in 40 genes and GO terms also referring to the metabolism of lipids and organophosphates, albeit with higher *p*-values.

### Metabolic pathways with prognostic relevance for MB patients

**Fig. 5:**
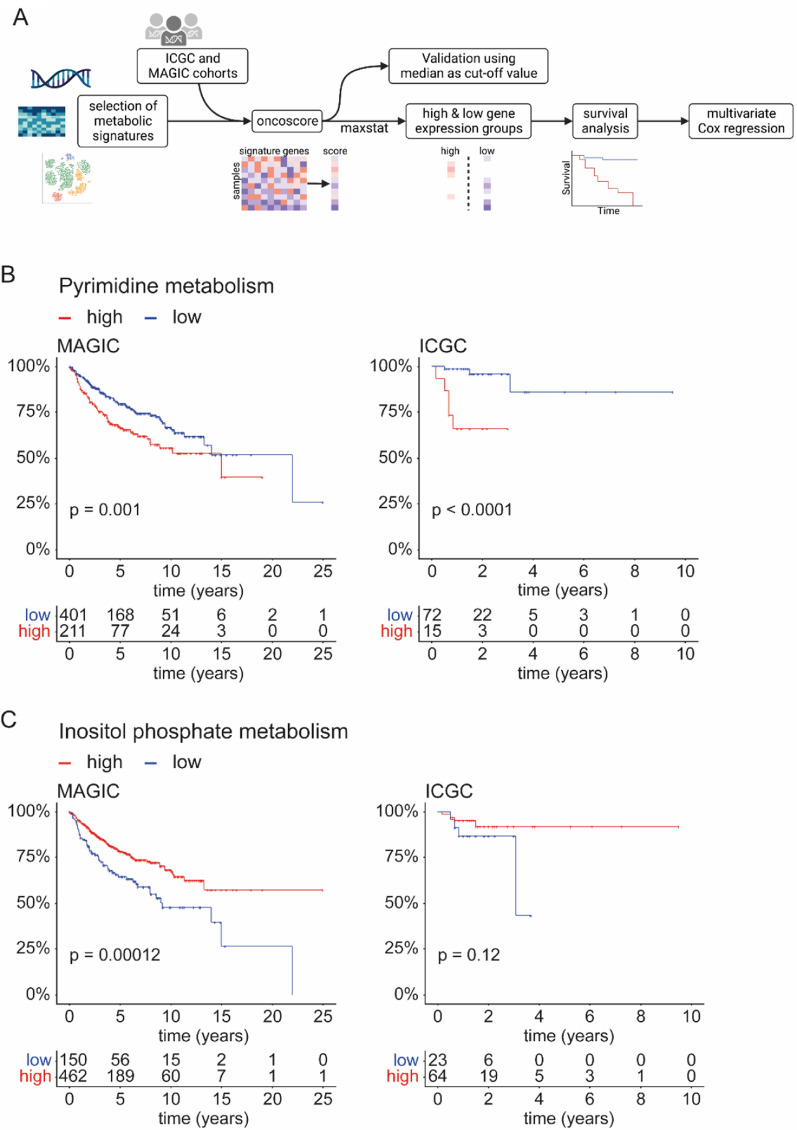
Nucleotide and IP metabolism are of prognostic relevance for MB patients. **A** Workflow showing all steps of the survival analysis using maximally selected rank statistics. The separation of ICGC and MAGIC cohorts into high and low gene expression groups has been performed using the R package maxstat as described in the Additional file 1. **B**, **C** Kaplan–Meier curves showing the overall survival of patients from MAGIC and ICGC cohorts. Patients have been divided into two groups depending on RNA expression levels of genes involved in the metabolism of **B** pyrimidines or **C** inositol phosphates. Log-rank test was used to calculate *p*-values, and *p* < 0.05 was considered significant. The flowchart was created using Biorender.com

Several metabolic pathways repeatedly appeared aberrant throughout analyses, e.g. metabolism of lipids, IP compounds, and nucleotides. For this reason, we evaluated how expression levels of genes involved in these pathways correlate with patients’ outcomes, including all MB groups. A detailed description of the analytical steps carried out can be found in the Additional file 1. Briefly, metabolic gene signatures from Rosario et al. [26] were selected based on previous results. For every sample and every signature, gene expression values were summarised into an oncoscore. Maximally selected rank statistics [39] were performed to identify the optimal cut-off value for each score to divide both cohorts into high and low gene expression groups. Subsequently, these groups were compared regarding patients’ prognoses (Fig. 5A). Survival analyses revealed high gene expression in pyrimidine metabolism being associated with an unfavourable and IP metabolism with a better prognosis (Fig. 5B, C). The latter was only observed in the MAGIC cohort. Purine metabolism showed a similar trend to pyrimidine metabolism, highlighting the relevance of deregulated nucleotide metabolism (Additional file 1: Fig. S14A). However, grouping by maximally selected rank statistics did not reach significance in this case.

Next, multivariate Cox regression analysis was conducted to determine the independence of identified risk factors. Both cohorts were stratified for MB groups, and the influence of high gene expression in pyrimidine and IP metabolism on patients’ overall survival compared to the respective low gene expression groups was tested. Increased expression of genes involved in pyrimidine metabolism was associated with a significantly higher hazard ratio in both cohorts. In contrast, IP metabolism significantly reduced the hazard ratio in the MAGIC cohort (Additional file 1: Fig. S13A, B).

These results imply that genes essential for pyrimidine and IP metabolism are relevant prognostic risk factors in MB.

## Discussion

Many factors influence cell metabolism, leading to diverse metabolic phenotypes even within one tumour entity [11]. While there have been other studies analysing metabolism in MB, we are only beginning to understand metabolic heterogeneity between MB groups [40]. Park et al., for example, established prognostically relevant metabolic pathways for SHH, G3 and G4 MB by exploring RNA expression data of the MAGIC cohort [16]. Comparable to their work, this study demonstrates striking metabolic differences on a transcriptomic level among MB groups. However, by starting with an unsupervised approach, we aimed to take differences also within established groups into account. Further, considering cancer metabolism to be a multi-faceted concept, we extended our analysis by also including scRNA-seq as well as nDNA data. We focused our study on G3/G4 MB because the relevance of biological processes concerning overlaps and differences in these groups has not yet been definitively clarified.

G3/G4 MB are separated into three metabolic clusters with significant differences in patient survival. In terms of intracellular effectors of cell metabolism, oncogenic driver events are known to play a central role in shaping the metabolic phenotype [9, 11]. It can therefore be assumed that varying developmental pathways, e.g. WNT and SHH [4], or the oncogene *MYC *[10] likely contribute to the metabolic clustering identified in this study. Likewise, our data showed a clear overlap of high-risk metabolic clusters with published G3/G4 consensus methylation subgroup II and correspondingly a high fraction of *MYC*-amplified samples [2]. Consistent with our results, MB samples with *MYC* amplification or overactivation separated from others when exploring proteomic data [41] and exhibited upregulation of genes related to OXPHOS, ribosomal genes and nucleotide metabolism in previous studies [42–44]. Although *MYC* is associated with a glycolytic phenotype [10, 43–45], stimulation of aerobic ATP production and anabolic pathways for biomass generation under normoxic conditions has been described [11]. In accordance, a recent review outlining metabolic characteristics of MB established thus far concluded that aerobic glycolysis and OXPHOS might be coexistent in MB [40]. Furthermore, the electron transport chain is closely linked to the metabolism of nucleotides [18]. The latter has been considered essential for proliferating cancer cells as it provides components for synthesising DNA, RNA and other macromolecules [18, 46]. These findings are in agreement with those obtained by Park et al., stating that the pentose phosphate pathway, which is also relevant to nucleotide metabolism and provides macromolecules for numerous metabolic pathways [47], is of prognostic importance in G3 MB [16].

In contrast, standard-risk clusters of G3/G4 MB exhibit upregulated metabolism of IP compounds. Earlier, deregulated phosphoinositol metabolism was observed in a subgroup of G4 MB, which responded well to combination therapy of IP6 and cisplatin [48]. It might be interesting to examine whether IP6 also benefits G3 patients exhibiting an upregulation of genes involved in IP metabolism, e.g. I_G3/4.3 and M_G3/4.3 (Fig. 2C, F).

On top of that, we demonstrated that frequently mutated metabolic genes play a central role in MB development and were associated with lipid and IP metabolism. In line with this observation, Sinkala et al. explored genomic variants in over 10.000 patients with 32 different types of cancer and outlined that highly mutated metabolic genes in tumours are often involved in lipid metabolism and have a well-known role in carcinogenesis [49].

Concerning cell-extrinsic influences, the TME determines tumour metabolism by causing changes in the metabolic milieu and competition for scarce nutrients [12]. Similar to the heterogeneity observed across malignant cells, the metabolism of immune cells can vary depending on numerous factors, including cell type and activation [29]. On the one hand, this is consistent with data obtained in our MCP counter analysis highlighting significant differences in TME cell populations across bulk RNA clusters, which may contribute to their metabolic phenotype. On single-cell RNA level, most TME cell types exhibited distinct differences in the expression of metabolic genes, forming separate clusters in both cohorts. Beyond that, our analysis emphasised the effect of intertumoral differences on metabolism across clusters identified in bulk RNA analysis. Although previous studies by Gwynne et al. identified pyrimidine metabolism as a characteristic of a particular tumour cell type in G3 MB [50], gene signatures from metabolic clusters did not highlight different cell types in the scRNA data examined here.

It has become clear that varying metabolic pathways have prognostic value in cancer [51, 52]. We provide evidence that distinct pathways are associated with the outcome of patients suffering from MB. Upregulated pyrimidine metabolism correlates with a significantly decreased overall survival, while IP metabolism gene expression comes with a better prognosis. These results were confirmed by stratified multivariate Cox regression analysis. Regarding the ICGC cohort, the results of this analysis might be limited by the small number of samples with available clinical data. On the one hand, our findings agree with Peng et al. [52], suggesting a correlation between nucleotide metabolism and an unfavourable prognosis. A positive correlation was also shown between lipid metabolism and prolonged survival. Moreover, high expression of *DHFR* and *TYMS*, involved in nucleotide synthesis, is associated with poor prognosis in G4 MB [16]. However, regarding IP metabolism, the aforementioned G4 MB subgroup was associated with decreased survival compared to control MB [48], and low expression of *INPP5E*, a phosphatase involved in the metabolism of IP, has been described to correlate with a better prognosis [53]. One possible explanation for this may be the confinement of these studies to only one gene or one MB subgroup. Further studies are needed for a deeper understanding of IP metabolism in MB.

There are likely other factors influencing metabolism, which we cannot depict on a genomic and transcriptomic level. However, a strong relationship between gene expression and metabolite profiling data has been reported in the literature [52], implying that transcriptomic analysis is a suitable tool for exploring metabolic phenotypes.

In the context of the growing body of literature drawing attention to various aberrations in tumour cell metabolism, the idea of exploiting oncometabolism as a potential therapeutic target appears more and more compelling [54]. In the past, combinatorial therapies, including the pyrimidine antimetabolite gemcitabine or inhibition of DHODH, a key enzyme in de novo pyrimidine synthesis, yielded promising results in preclinical studies of *MYC*-amplified G3 MB [50, 55–57]. However, the metabolic flexibility of cancer cells and the need for therapies sparing surrounding non-malignant tissue pose a great challenge. In a recent review, the authors drew the conclusion that the effective use of metabolic inhibitors together with standard therapeutic options would necessitate vigorous screening to identify metabolic vulnerabilities downstream of established driver mutations and patients who might profit from these [58]. By comparing MB groups and subgroups under different aspects, our study aims to contribute to reaching this overarching goal.

## Conclusions

In summary, this study unravelled apparent differences in cell metabolism between MB subgroups. We established the metabolism of nucleotides and inositol phosphate compounds to be frequently deregulated in MB and of prognostic relevance for patients. Our results broaden the current understanding of intertumoral heterogeneity in MB and may pave the way for future studies on metabolism-targeted therapies in this tumour entity.

## Supplementary Information

**Additional file 1: Supplementary materials.** Supplementary materials include supplementary methods, references and the Supplementary Figures S1–S15.

**Additional file 2: Table S1.** Clinical data of samples from ICGC and MAGIC cohorts explored within the scope of our bulk RNA analysis. PFS = progression-free survival, OS = overall survival, M-stage = tumour dissemination stage.

**Additional file 3: Table S2.** Statistical analysis of clinical data from the ICGC cohort based on bulk RNA clustering is shown. Page one lists the results when comparing all ICGC clusters except for the excluded samples. Page two lists the results when comparing only I_G3/4.1, I_G3/4.2 and I_G3/4.3. The parameters age (continuous), progression-free and overall survival, M-stage, gender, age (paediatric vs adult), recurrence, and death cases have been tested, and the results are shown from top to bottom. PFS = progression-free survival, OS = overall survival, M-stage = tumour dissemination stage, nSample = number of samples tested, SD = standard deviation.

**Additional file 4: Table S3.** Complete list of GO terms of genes unique to ccmGDB and Rosario et al.

**Additional file 5: Table S4.** A detailed collection of DEGs and analysed pathways from bulk RNA analysis. For both cohorts, a complete set of DEGs when comparing all metabolic clusters and when comparing only G3/G4 clusterare provided. The according IPA canonical pathways and results from Metascape analyses are listed behind the according set of DEGs. Moreover, gene signatures resulting from this analysis and utilised for constructing the Sankey plot and scRNA-seq have been added.

**Additional file 6: Table S5.** Supplementary table 5 shows detailed results of the MCP-counter analysis. Abundance scores for ten different cell types across all included samples from ICGC and MAGIC cohorts are provided. Kruskal-Wallis and Wilcoxon rank-sum tests have been performed to test for statistical significance of observed differences.

**Additional file 7: Table S6.** Technical details on the sequencing runs of the six MB samples used for scRNA-seq analysis. For both scRNA-seq cohorts, the number of cells per patient and cluster is shown for the regular UMAP based on all genes and the UMAP based solely on metabolic genes. For the RMB cohort, MB group and subgroup affiliations are derived from Riemondy et al. Furthermore, a list of marker genes for cells from the TME has been provided. Lists of DEGs and analysed pathways from scRNA-seq analyses described in the Additional file 1 have been added for both scRNA-seq datasets.

**Additional file 8: Table S7.** Mutated metabolic genes detected within the scope of the nDNA analysis and GO terms of the according genes. Pages one and two refer to genes detected using the list of metabolic genes from ccmGDB. Results of the second version based on genes from Rosario et al. are listed on pages three and four.

**Additional file 9: Table S8.** Further details on statistical analysis using maximally selected rank statistics. On page one, all ten metabolic pathways explored are shown. Cut-off values calculated by the R package maxstat and *p*-values for the according cut-off value and for the Kaplan-Meier curves are depicted for MAGIC and ICGC cohorts. Here, p<0.05 was considered significant. The following pages list detailed information on RNA expression values, oncoscores and maxstat grouping for pyrimidine metabolism, inositol phosphate metabolism, purine metabolism, oxidative phosphorylation and pyruvate metabolism separated by cohort. *p*-val = *p*-value, KM = Kaplan-Meier curve, IP = inositol phosphate, OXPHOS = oxidative phosphorylation.

## Data Availability

RNA expression values of ICGC cohort were downloaded from the European Genome-phenome Archive (https://ega-archive.org/; dataset ID EGAD00001003279). Genomic mutations in samples from ICGC cohort were previously published by Northcott et al. [19]. Microarray data from MAGIC cohort analysed throughout this study were obtained from Gene Expression Omnibus GEO (https://www.ncbi.nlm.nih.gov/geo; accession number GSE85217). Single-cell RNA sequencing data from MSMB cohort supporting the conclusions of this article are available on Gene Expression Omnibus GEO (https://www.ncbi.nlm.nih.gov/geo; accession number GSE212559). Published scRNA-seq data were retrieved from Gene Expression Omnibus GEO (https://www.ncbi.nlm.nih.gov/geo; accession number GSE155446).

## Abbreviations

ccmGDB: Cancer cell metabolism gene database
CI: Confidence interval
CPO: Cell projection organisation
DEG: Differentially expressed gene
ECM: Extracellular matrix
EMT: Epithelial–mesenchymal transition
ETC: Electron transport chain
G3: Group 3
G4: Group 4
GO: Gene Ontology
HR: Hazard ratio
INDEL: Insertions and deletions
IP: Inositol phosphate
KM: Kaplan–Meier curve
logFC: Log-fold change
MB: Medulloblastoma
MBNOS: MB not other specified
MSMB: ScRNA-seq cohort of six samples from Münster
M-stage: Tumour dissemination stage
nDNA: Nuclear DNA
nSample: Number of samples tested
OS: Overall survival
OXPHOS: Oxidative phosphorylation
PCA: Principal component analysis
PFS: Progression-free survival
PPP: Pentose phosphate pathway
*p*-val: *P*-value
RMB: ScRNA-seq data published by Riemondy et al.
scRNA-seq: Single-cell RNA-sequencing
SD: Standard deviation
SNVs: Single nucleotide variants
TME: Tumour microenvironment
tpm: Tags per million
WGS: Whole-genome sequencing
wt: Wildtype

## Declarations

### Previous publications

Parts of the content of this article have previously been presented at the 20^th^ International Symposium on Pediatric Neuro-Oncology (ISPNO 2022). The abstract published within the scope of this presentation has been included in the list of references [59].
