# Supplementary material for: Group-specific cellular metabolism in Medulloblastoma
Source: J Transl Med. 2023 Jun 5;21:363. doi: 10.1186/s12967-023-04211-6 (PMC10242934; doi:10.1186/s12967-023-04211-6)
Supplement: Supplementary file 1 — Additional file 1: Supplementary materials. Supplementary materials include supplementary methods, references and the Supplementary Figures S1–S15. [file 12967_2023_4211_MOESM1_ESM.pdf]

Supplementary materials for

Group-specific cellular metabolism in Medulloblastoma

Viktoria LE Funke, Carolin Walter, Viktoria Melcher, Lanying Wei, Sarah Sandmann, Marc Hotfilder, Julian Varghese, Natalie Jäger, Marcel Kool, David TW Jones, Stefan M Pfister, Till Milde, Martin Mynarek, Stefan Rutkowski, Jochen Seggewiss, Daniela Jeising, Flavia W de Faria, Thorsten Marquardt, Thomas K Albert, Ulrich Schüller, Kornelius Kerl

Table of Contents

1. Supplementary Methods 2

1.1. Bulk RNA-seq analysis of ICGC and MAGIC cohorts' metabolism genes 2

1.1.1. ICGC 2

1.1.2. MAGIC 3

1.1.3. Statistical Analysis 3

1.1.4. Functional analysis 4

1.2. Single-cell RNA-seq analysis 5

1.3. Nuclear DNA analysis 9

1.4. Maxstat analysis and survival curves 9

2. Supplementary figures 11

3. Supplementary References 37

1. Supplementary Methods

1.1. Bulk RNA-seq analysis of ICGC and MAGIC cohorts' metabolism genes

1.1.1. ICGC

Normalised expression values (tags per million, tpm) of 148 medulloblastoma RNA-seq samples were available from the ICGC database (https://ega-archive.org/; dataset ID EGAD00001003279). Nine samples were excluded as potential outliers based on RNA expression patterns and a principal component analysis (PCA) plot (Additional file 1: Fig. S1A). 139 remaining samples were used as input for an unsupervised clustering with R v4.0.5(1) and the R package pheatmap(2). The input dataset was limited to a set of 2,071 metabolism-related genes(3), and the clustering method was set to "complete". Silhouette method was conducted using R package cluster(4) to determine an optimal number of clusters. In addition to the silhouette method, we considered the previous classification of samples, visual inspection of the dendrogram splitting and corresponding heatmap patterns. Despite an optimal number of three clusters, seven clusters were created in total to be able to focus on differences between established MB groups. Further data pre-processing and annotation were conducted with basic R functions. For each cluster, differentially expressed genes (DEGs) were calculated with DESeq2(5), and Kaplan-Meier curves were plotted for all samples with available survival data (n=87) with the R package survminer(6) and default parameters. One cluster was excluded after DEG analysis. In the initial quality control described above, it was part of a group of borderline samples (Additional file 1: Fig. S1A), which were not excluded to avoid a further reduction of cohort size. As no matching cluster was found in the larger MAGIC validation cohort, this cluster was excluded from further

analysis. Due to the unavailability of clinical data for most samples of this cluster, the according cluster had not been included in the statistical analysis.

1.1.2. MAGIC

Pre-processed normalised log2 gene expression values for 763 medulloblastoma RNA microarray samples were downloaded from the MAGIC cohort dataset GSE85217 and restricted to the 2,071 chosen metabolism-related genes that were used for the preparation of the ICGC cohort. A heatmap was created with pheatmap using the ward.D clustering function. The definition of clusters was implemented considering the results of the silhouette method, gene expression patterns, and the heatmap's dendrogram splitting in line with the ICGC cohort, resulting in six clusters in total. DEGs between clusters were calculated with the R/Bioconductor package limma(7). As with the ICGC cohort, Kaplan-Meier curves were plotted in R for samples with fully available survival data (n=612) using survminer and default parameters.

For both ICGC and MAGIC cohorts, bulk RNA heatmaps were also constructed using a second set of 1,771 genes compiled from metabolic pathway signatures from Rosario et al.(8) to validate our findings. Genes encoded by the mitochondrial DNA were excluded from the validation of bulk RNA and nDNA analyses but are still part of the OXPHOS gene signature used in maxstat analysis described below. Intersect function in R was employed to calculate overlapping and unique elements of both gene lists. Unique genes were further characterised by utilising the ToppGene Suite (https://toppgene.cchmc.org/).

1.1.3. Statistical Analysis

ICGC MB metadata was extracted for the parameters age (continuous and paediatric vs adult), progression-free (PFS) and overall survival (OS), tumour dissemination stage (M-stage), gender, recurrence and death cases. R was used to conduct Shapiro

tests for normality per cluster and parameter. Kruskal-Wallis and pairwise Wilcoxon rank-sum tests were applied to test for significant differences in age (continuous), PFS and OS, and M-stage. A Chi-square test was used to test variables gender, age (paediatric vs adult), recurrence and death cases for independence, while a pairwise Fisher test was conducted to compare the observed proportions of two clusters. Additionally, for age (paediatric vs adult), recurrence and death cases, observed proportions of cases were compared to the average proportion of the dataset with exact binomial test (for $n<30$) and I-sample proportions test with continuity correction (for $n \geq 30$). All statistical tests were calculated with basic R functions (significance level 0.05, adjustment method Benjamini-Hochberg).

1.1.4. Functional analysis

For all clusters (bulk RNA and single-cell RNA), IPA canonical pathway analysis of DEGs was performed. Due to the uneven distribution of high and low expressed DEGs among clusters (Additional file 1: Fig. S3B), analysis was limited to upregulated DEGs (logFC>0). Resulting pathways were sorted by p-value and filtered for relevant pathways based on IPA classification (focus on metabolism) and existing literature.

Bulk RNA analysis was repeated employing Metascape (version: v3.5.20230101)(9). In line with the previous approach, two sets of DEG lists were explored for both cohorts. Highly expressed DEGs (logFC≥1) comparing all clusters were examined for ICGC and MAGIC cohorts and comparing only G3/G4 clusters for the MAGIC cohort (Additional file 1: Fig. S4). The restriction to DEGs with a logFC≥1 was done due to a limitation to the number of genes Metascape can analyse simultaneously. When only ICGC G3/G4 clusters are compared, only a few DEGs meet this criterium for I_G3/4.3. Therefore a comparative analysis of G3/G4 clusters was replaced by a separate

analysis of the according DEG lists of I_G3/4.1, I_G3/4.2 and I_G3/4.3 (Additional file 1: Fig. S5). All upregulated DEGs (logFC>0) were considered for the latter.

Computational deconvolution of bulk RNA samples from both cohorts was conducted with R v4.1.3(10) and MCP-counter version 1.2.0(11). For both cohorts, the abundance of cells from the tumour microenvironment (TME) was compared across all clusters and only G3/G4 clusters. Kruskal-Wallis and Wilcoxon rank-sum tests were used to assess the significance of discovered differences. P-values were adjusted for multiple comparisons using Bonferroni correction.

Sankey plots were constructed for DEG comparison utilising the R-package networkD3(12). DEGs were sorted by logFC (high to low). For Fig. 2G, the top 250 unique, upregulated DEGs of every cluster have been included. A second plot was constructed using the top 250 upregulated DEGs in general (Additional file 1: Fig. S3C).

1.2. Single-cell RNA-seq analysis

Within the scope of the single-cell RNA-seq (scRNA-seq) analyses, data from two cohorts of MB patients were examined. First, we explored data from six human MB samples treated in Münster, referred to as MSMB. Second, we validated our findings using scRNA-seq data of 28 paediatric MB patients (abbreviated as RMB) published by Riemondy et al.(13).

1.2.1. MSMB

scRNA-seq analyses were conducted with the 10x Genomics CellRanger(14) and Seurat(15) pipelines, respectively. For the regular human medulloblastoma scRNA-seq clustering, six human MB samples were pre-processed with CellRanger v3.0.2. After converting the raw 10X data to fastq format with CellRanger’s mkfastq, the data

were aligned against the human reference transcriptome GRCh38 with the CellRanger count function using default values and the filtered feature matrices were tested for basic quality statistics with CellRanger's web summary function. Samples with a sequencing saturation of <50% after the first sequencing run were re-sequenced when possible.

The CellRanger data were subsequently processed with R v4.0.5 and Seurat v4.0.5. Seurat objects were created per sample with a minimum of three cells and a minimum feature number of 200; furthermore, only cells with less than 25% of mitochondrial genes were considered further. Additionally, outlier cells with a high nCount_RNA value were considered to be doublets and removed accordingly; the threshold for removal varied per sample (7,000 to 80,000, mean=26833.33).

All filtered samples were then processed with Seurat’s SCTransform function and default parameters. Dimensionality reduction was conducted per PCA, integration anchors were identified with Seurat, and the dataset was integrated using the previously calculated anchorset to remove batch effects.

Based on this integration, a clustering was created with Seurat’s FindCluster function using a resolution parameter of 0.5 and default parameters otherwise. UMAPs were used to visualise cluster and sample distributions. Differential genes were calculated with Seurat’s FindMarkers function for each cluster and analysed using QIAGEN IPA. Annotation of G3/G4 cell clusters was carried out by cell numbers (>75% cells of one MB group) and published gene signatures(16,17). Cell types of the TME were assigned by employing their respective marker genes(13,18–23) and considering IPA canonical pathways. For metabolic gene signatures, DEGs of bulk RNA clusters were sorted by logFC (high to low). Top 50 upregulated DEGs were included per cluster. Overlap of signatures (Additional file 1: Fig. S9A) was calculated using intersect function in R to

determine whether each signature unequivocally represents its metabolic bulk RNA cluster. The analysis was conducted with DEGs of both cohorts with two sets of DEG lists: one cluster against all others and comparing only G3/G4 clusters, as shown in Additional file 1: Fig. S9. Chosen marker genes and gene signatures were visualised by creating feature plots and violin plots. For metabolic gene signatures, gene expression values illustrated by the UMAP legend were aligned for each set of DEG lists to compare signatures directly. Seurat's cell cycle scoring approach was used based on a marker gene set by Tirosh et al.(24) to group the integrated dataset into cell cycle phases, and CNV candidates were determined with the R package CONICSmat v0.0.0.1(25).

For a second, separate clustering, the original MB sample data was restricted to a predefined set of 2,071 metabolic target genes, and filter criteria were relaxed to a minimum feature number of 20. The remaining Seurat workflow was conducted as described above. For both clusterings, further data exploration and analyses were conducted with the R/Bioconductor package iSEE v2.6.0(26). For each cluster, IPA canonical pathway analysis was performed comparing upregulated DEGs, in general, and unique upregulated DEGs. Cluster annotations have been based on cell numbers, manual evaluation of all resulting canonical pathways and of highly expressed unique DEGs (logFC≥1) by literature. Prominent metabolic canonical pathways from cluster 14 (classified as macrophages) were further validated by analysing unique DEGs (logFC>0) using Metascape. Annotations were transferred from metabolic set to regular clustering on cell level (Additional file 1: Fig. S10H) with basic R functions. The results for the TME and cycling cells are depicted in Fig. 3F. DEG lists, canonical pathways of all clusters and results from Metascape analysis of macrophage cluster

are provided in Additional file 7: Table S6. In this study, “unique DEG” refers to comparing upregulated DEGs if not specified otherwise.

For Additional file 1: Fig. S10D, genes of significant canonical metabolic pathways were grouped depending on metabolic categories stated in IPA. Genes belonging to multiple categories were counted once per category. Categories containing more than 20% of all metabolic genes classified for the according cluster are presented in Additional file 1: Fig. S10E-G.

1.2.2. RMB

Processed scRNA-seq data published by Riemondy et al. were retrieved from Gene Expression Omnibus GEO (https://www.ncbi.nlm.nih.gov/geo; accession number GSE155446). The downloaded dataset included 30 samples from 28 paediatric MB patients. Subsequently, the creation of Seurat objects for both the regular clustering based on all genes and the metabolic UMAP were performed as described above. Regarding the standard clustering, the annotation of gene expression signatures representing MB groups relied mainly on marker genes instead of cell numbers due to a more substantial mixing of samples from different groups. Similarly, cell types of the TME and metabolic signatures were annotated in the standard UMAP based on the marker genes mentioned above.

For characterisation of the metabolic UMAP, DEGs were calculated with R v4.1.3 and analysed in line with MSMB cohort. Classification of specific DEGs in metabolic categories as well as the transfer of clustering in the metabolic UMAP to the regular analysis are shown in Additional file 1: Fig. S11.

1.3. Nuclear DNA analysis

We searched previously published DNA variants of the ICGC cohort(27) for mutations in metabolic genes from ccmGDB in samples of all 491 patients from ICGC cohort. The number of mutated samples was counted per aberrant gene. If different qualities of variants were present (single nucleotide variants, insertions/deletions, etc.), sample count was split up depending on variant composition in that sample (Fig. 4B). Functional analysis of mutated genes was performed using the ToppGene Suite.

In accordance with our bulk RNA analysis, we validated our findings using the second set of metabolic genes introduced above(8). Classification of single nucleotide variants was retrieved from Northcott et al.(27) for both sets of metabolic genes.

1.4. Maxstat analysis and survival curves

A set of metabolic gene signatures derived from Rosario et al.(8) was chosen based on results from previous analyses and literature research. Normalised expression data for selected signature genes were extracted from the ICGC and MAGIC cohort. For each signature, the mean expression of the signature's genes was calculated per sample and subsequently normalised to [0,1] with R. This procedure was repeated for every sample of ICGC and MAGIC cohorts with available RNA expression values and survival data regardless of MB group (ICGC n=87; MAGIC n=625) to create a signature-specific "oncoscore" for each dataset. In line with the statistical analysis described above, the cluster excluded after the introduction of the MAGIC cohort was not included in this survival analysis.

The R package maxstat (parameters: smethod = "LogRank", pmethod = "condMC", minprop = 0.2) was then used to perform a maximally selected rank statistics analysis

on the survival data and oncoscore in order to split the chosen RNA cohort into a "high" and "low" scoring group according to maxstat's optimised threshold value(28,29). Survival curves were computed using survfit function(30) and then plotted with the R package survminer(6) and default parameters. Metabolic signatures, oncoscores, cut-off values and statistical data are provided in Additional file 9: Table S8.

Multivariate cox regression analysis was performed utilising the R packages survival(30) and survminer(6). The regression models included 87 samples of ICGC and 612 samples of the MAGIC cohort, respectively, and evaluated the effect of the variables IP and pyrimidine metabolism as well as the MB groups. In MAGIC cohort, 13 samples were automatically excluded throughout the process due to missing data. For both metabolic pathways grouping by maxstat as "low gene expression" was used as the reference variable, and for the MB groups, WNT was considered the reference. The proportional hazard assumption was tested for a regression model including both metabolic pathways and using cox.zph function (survival package). Both cohorts were stratified for MB groups. Survminer's ggcoxdiagnostics function was employed to visualise dfbeta values in order to test for influential observations. Observations exceeding a cutoff of $2/\sqrt{n}$ were identified as potentially influential. Two samples causing influential observations in ICGC cohort were not excluded in order to avoid further reduction of cohort size and number of events. There were no influential observations in MAGIC cohort. Forest plots were constructed with the R package forestplot(31).

Repetition of survival analysis using the median oncoscore as cut-off value for the division of cohorts was conducted as a control. Samples with exact median oncoscore have consistently been assigned to the "high gene expression" group.

2. Supplementary figures

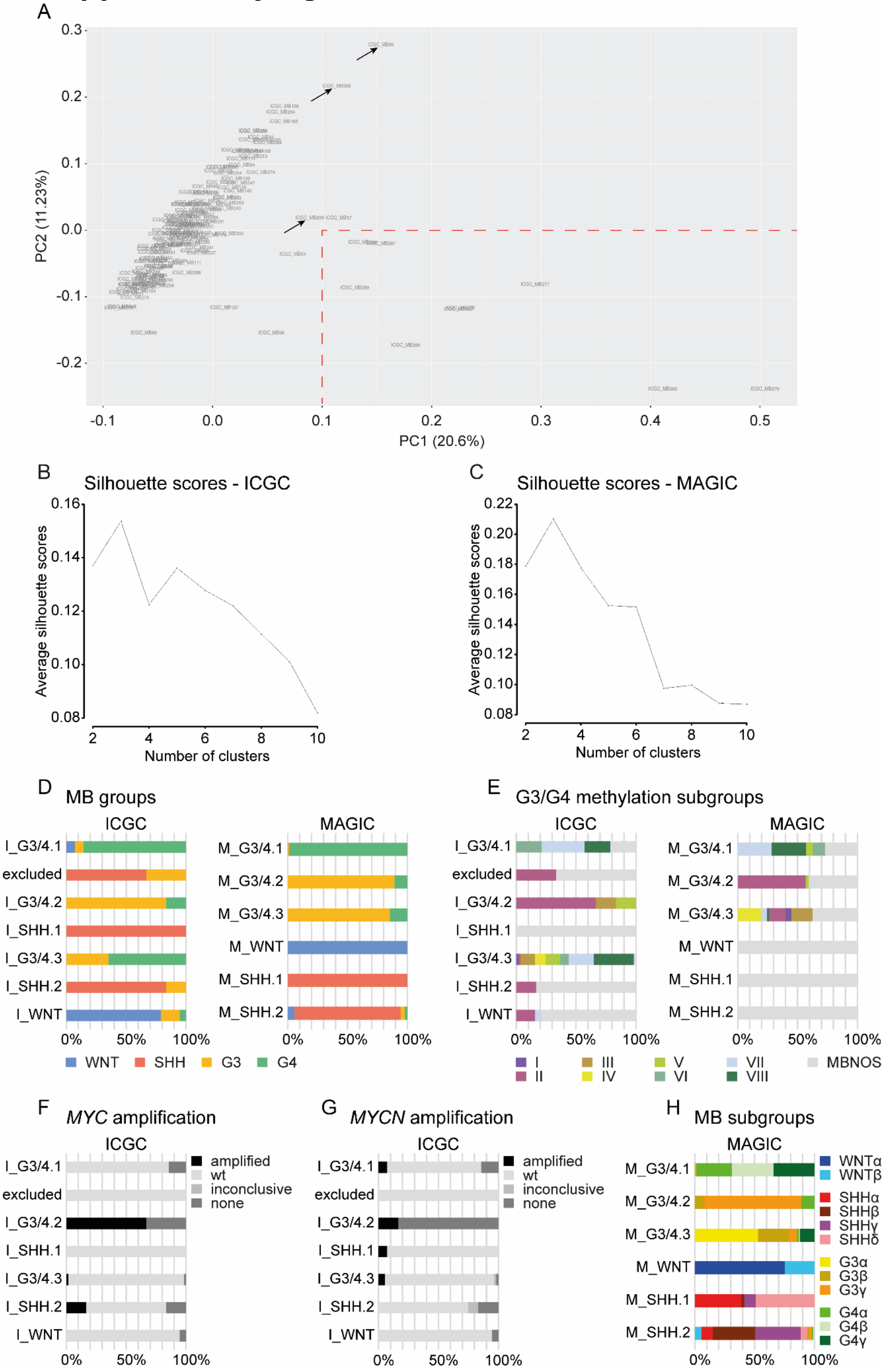

Fig. S1, related to Fig. 1: Characterisation of metabolic clusters

A) PCA plot showing all 148 samples from the ICGC cohort. Red dashed lines frame samples excluded during initial quality control. Black arrows highlight samples from the excluded cluster. B), C) Average silhouette scores for varying numbers of clusters for B) ICGC and C) MAGIC cohorts are depicted. Highest silhouette scores indicate an optimal number of three clusters for both cohorts. Stacked bar plots illustrating the distribution of D) established MB groups, E) G3/G4 methylation subgroups(32), F) MYC amplified samples, G) MYCN amplified samples and H) MB subgroups established by Cavalli et al.(33) across metabolic clustering identified in Fig. 1. G3 = Group 3; G4 = Group 4; MBNOS = MB not other specified; wt = wildtype

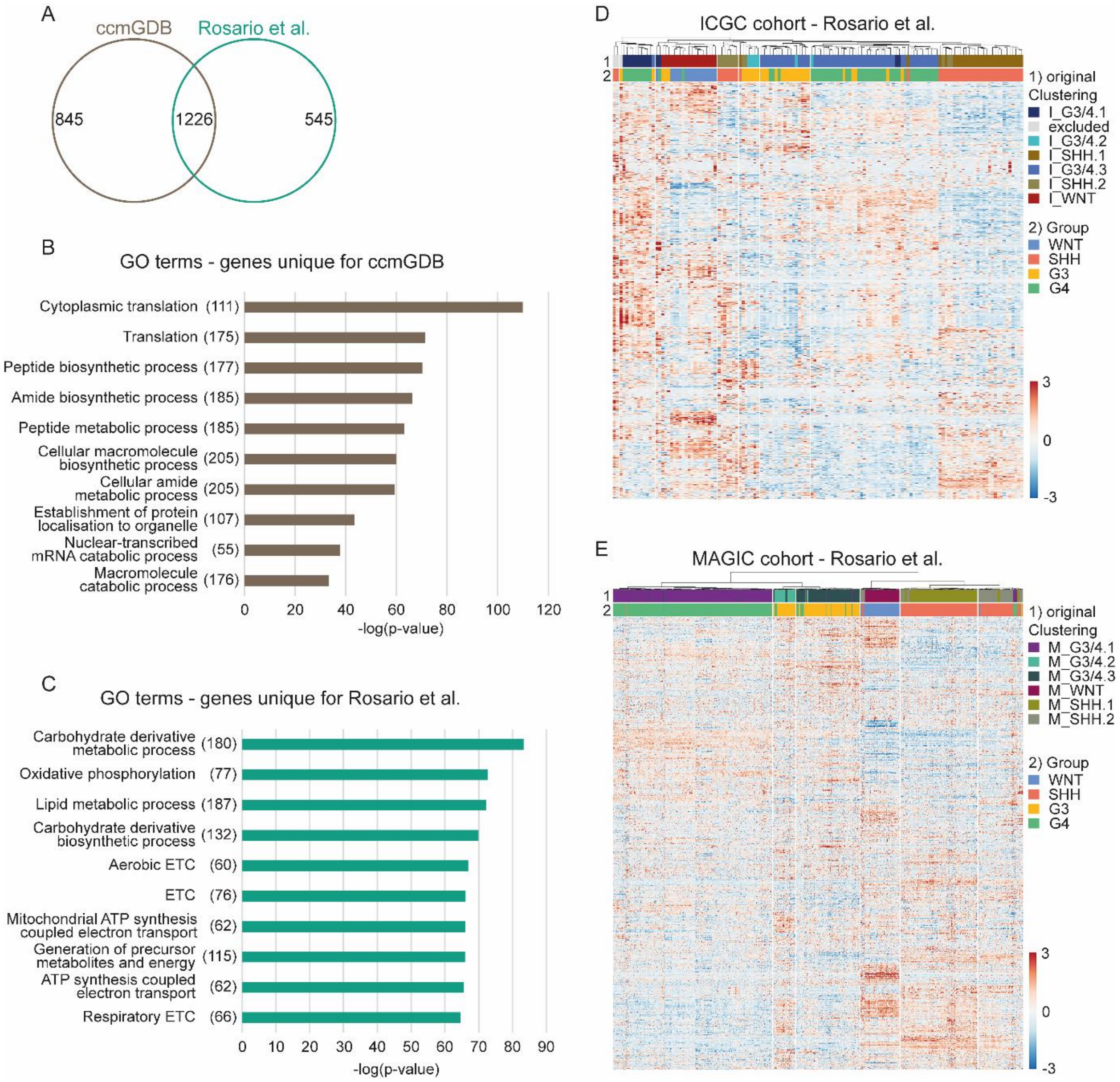

Fig. S2, related to Fig. 1: Metabolic clustering with metabolic genes from Rosario et al.

A) Overlap of the two gene lists used within the scope of the bulk RNA analysis is illustrated. ToppGene Suite has been employed to characterise genes which are unique to the gene list from B) ccmGDB and C) Rosario et al.(8). For every GO term, the number of genes matching the according pathway is shown in brackets. E) and D) show the results of unsupervised hierarchical clustering of samples from D) ICGC and E) MAGIC cohorts based on gene expression levels of the list derived from Rosario et

al. Affiliation of depicted samples to original metabolic clustering (Fig. 1A) and MB groups are shown from top to bottom for both cohorts. ETC = electron transport chain

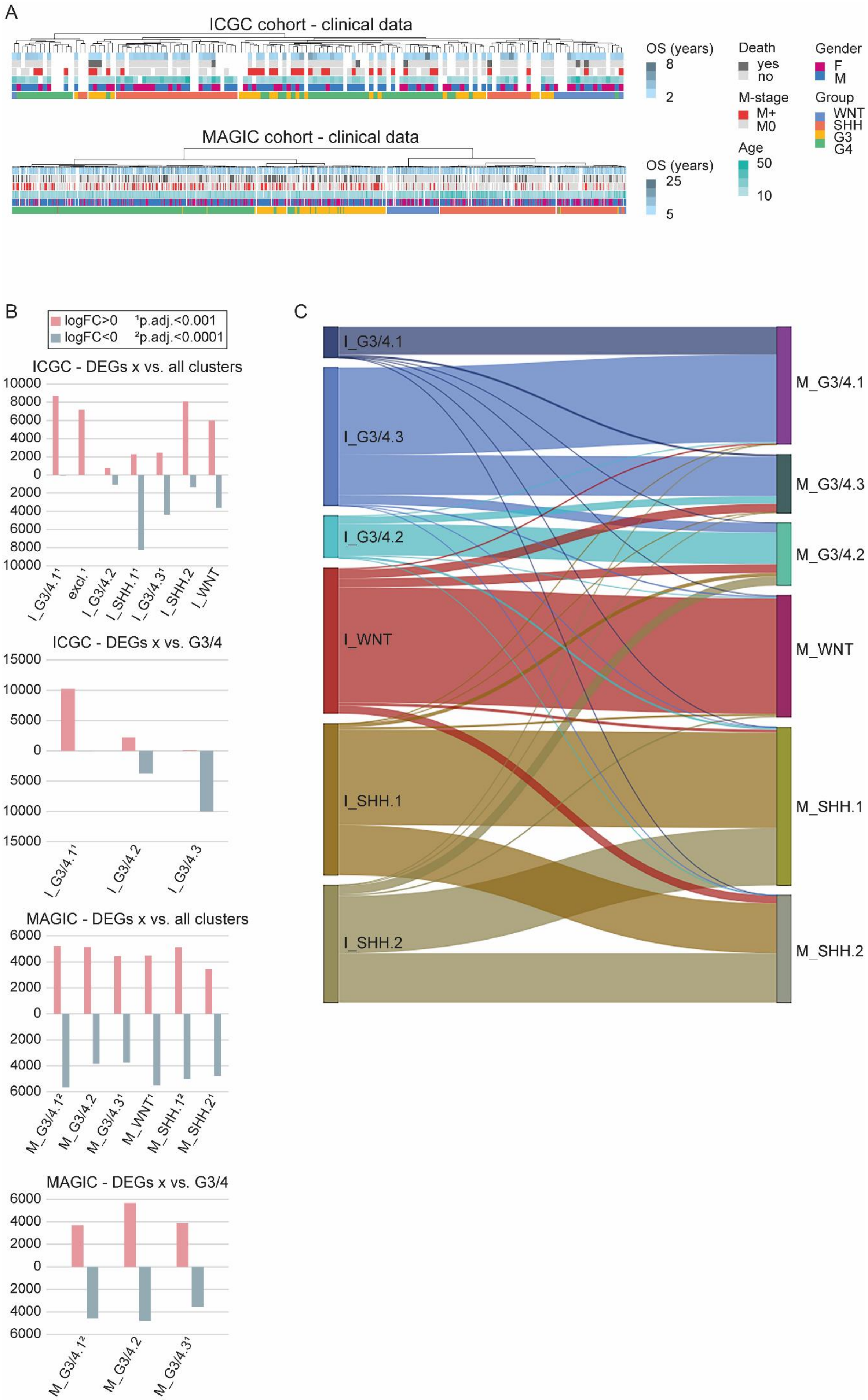
A
ICGC cohort - clinical data
MAGIC cohort - clinical data
OS (years)
8
2
OS (years)
25
5
Death
yes
no
M-stage
M+
M0
Age
50
10
Gender
F
M
Group
WNT
SHH
G3
G4
B
logFC>0
logFC<0
¹p.adj.<0.001
²p.adj.<0.0001
ICGC - DEGs x vs. all clusters
10000
8000
6000
4000
2000
0
2000
4000
6000
8000
10000
I_G3/4.1¹
excl.¹
I_G3/4.2
I_SHH.1¹
I_G3/4.3¹
I_SHH.2
I_WNT
ICGC - DEGs x vs. G3/4
15000
10000
5000
0
5000
10000
15000
I_G3/4.1¹
I_G3/4.2
I_G3/4.3
MAGIC - DEGs x vs. all clusters
6000
4000
2000
0
2000
4000
6000
M_G3/4.1²
M_G3/4.2
M_G3/4.3¹
M_WNT¹
M_SHH.1²
M_SHH.2¹
MAGIC - DEGs x vs. G3/4
6000
4000
2000
0
2000
4000
6000
M_G3/4.1²
M_G3/4.2
M_G3/4.3¹
C
I_G3/4.1
I_G3/4.3
I_G3/4.2
I_WNT
I_SHH.1
I_SHH.2
M_G3/4.1
M_G3/4.3
M_G3/4.2
M_WNT
M_SHH.1
M_SHH.2

Fig. S3, related to Fig. 1 and 2: Clinical data and functional analyses of ICGC and MAGIC

A) Colour bars outline the clinical characteristics of patients in both cohorts. Sample grouping reflects unsupervised hierarchical clustering, as presented in Fig. 1A. Data encompassing patient age, gender, overall survival, tumour dissemination stages and death cases were examined. For ICGC cohort, tumour dissemination stages M1, M2 and M3 (Additional file 2: Table S1) have been summarised as M+. Where no colour is shown, data were unavailable. OS = overall survival; M-stage = tumour dissemination stages

B) Bar graphs illustrating the number of genes for every set of DEGs employed for bulk RNA analyses. Lists of DEGs have been generated with p.adj. < 0.05 unless stated otherwise. C) Sankey plot similar to Fig. 2G but when considering the top 250 upregulated DEGs in general instead of focusing on unique genes.

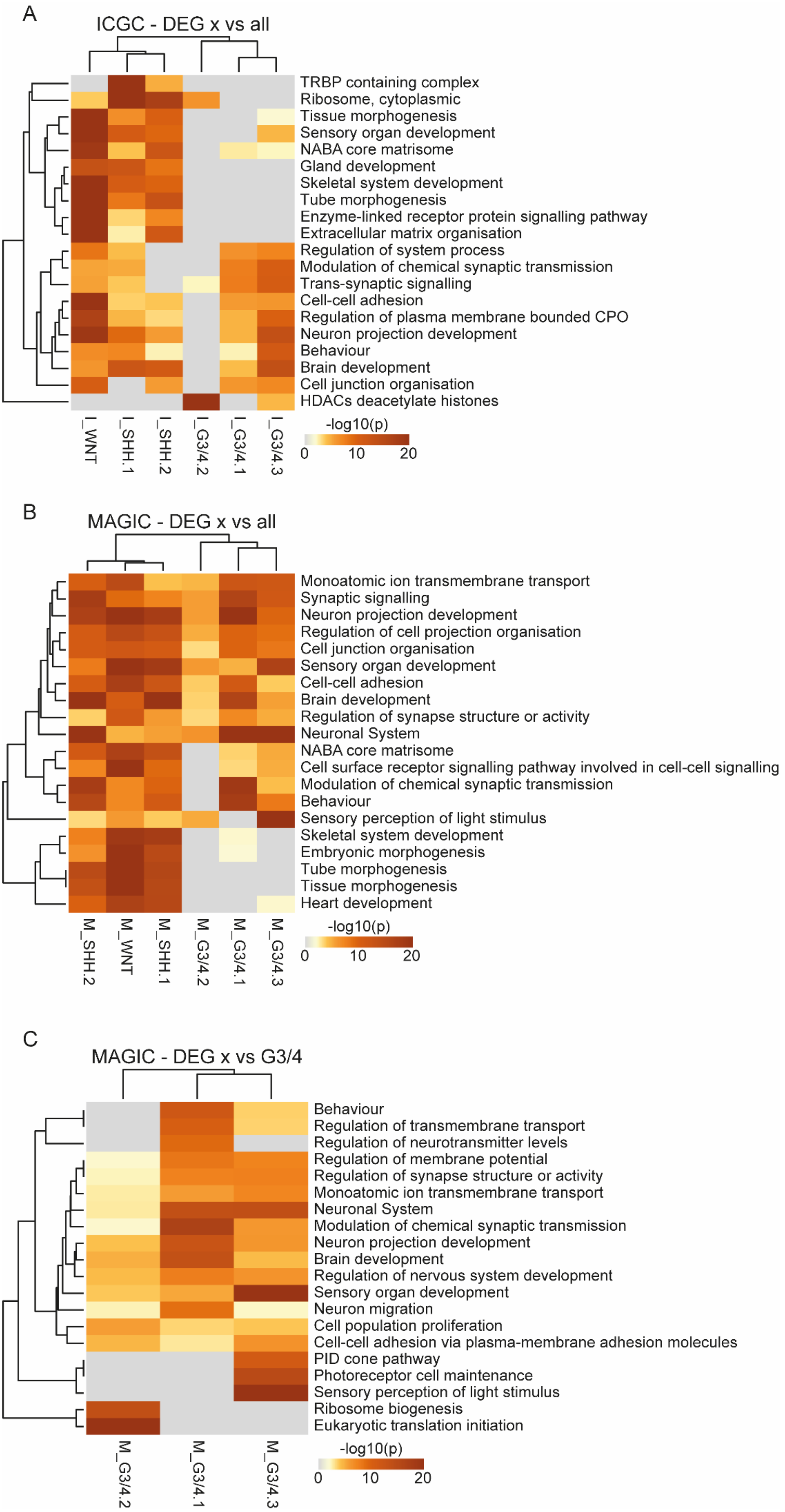

A
ICGC - DEG x vs all
TRBP containing complex
Ribosome, cytoplasmic
Tissue morphogenesis
Sensory organ development
NABA core matrisome
Gland development
Skeletal system development
Tube morphogenesis
Enzyme-linked receptor protein signalling pathway
Extracellular matrix organisation
Regulation of system process
Modulation of chemical synaptic transmission
Trans-synaptic signalling
Cell-cell adhesion
Regulation of plasma membrane bounded CPO
Neuron projection development
Behaviour
Brain development
Cell junction organisation
HDACs deacetylate histones
I_WNT
I_SHH.1
I_SHH.2
I_G3/4.2
I_G3/4.1
I_G3/4.3
-log10(p)
0
10
20
B
MAGIC - DEG x vs all
Monoatomic ion transmembrane transport
Synaptic signalling
Neuron projection development
Regulation of cell projection organisation
Cell junction organisation
Sensory organ development
Cell-cell adhesion
Brain development
Regulation of synapse structure or activity
Neuronal System
NABA core matrisome
Cell surface receptor signalling pathway involved in cell-cell signalling
Modulation of chemical synaptic transmission
Behaviour
Sensory perception of light stimulus
Skeletal system development
Embryonic morphogenesis
Tube morphogenesis
Tissue morphogenesis
Heart development
M_SHH.2
M_WNT
M_SHH.1
M_G3/4.2
M_G3/4.1
M_G3/4.3
-log10(p)
0
10
20
C
MAGIC - DEG x vs G3/4
Behaviour
Regulation of transmembrane transport
Regulation of neurotransmitter levels
Regulation of membrane potential
Regulation of synapse structure or activity
Monoatomic ion transmembrane transport
Neuronal System
Modulation of chemical synaptic transmission
Neuron projection development
Brain development
Regulation of nervous system development
Sensory organ development
Neuron migration
Cell population proliferation
Cell-cell adhesion via plasma-membrane adhesion molecules
PID cone pathway
Photoreceptor cell maintenance
Sensory perception of light stimulus
Ribosome biogenesis
Eukaryotic translation initiation
M_G3/4.2
M_G3/4.1
M_G3/4.3
-log10(p)
0
10
20

Fig. S4, related to Fig. 2: Comparative DEG analysis using Metascape

Heatmaps showing hierarchical clustering of significant pathways from DEG analysis with Metascape. Analysis has been performed using DEGs comparing all metabolic clusters for A) ICGC and B) MAGIC cohorts. C) The latter cohort's analysis has been repeated with DEGs, comparing only G3/G4 clusters. Only highly expressed DEGs (logFC≥1) have been considered for all three analyses. CPO = cell projection organisation

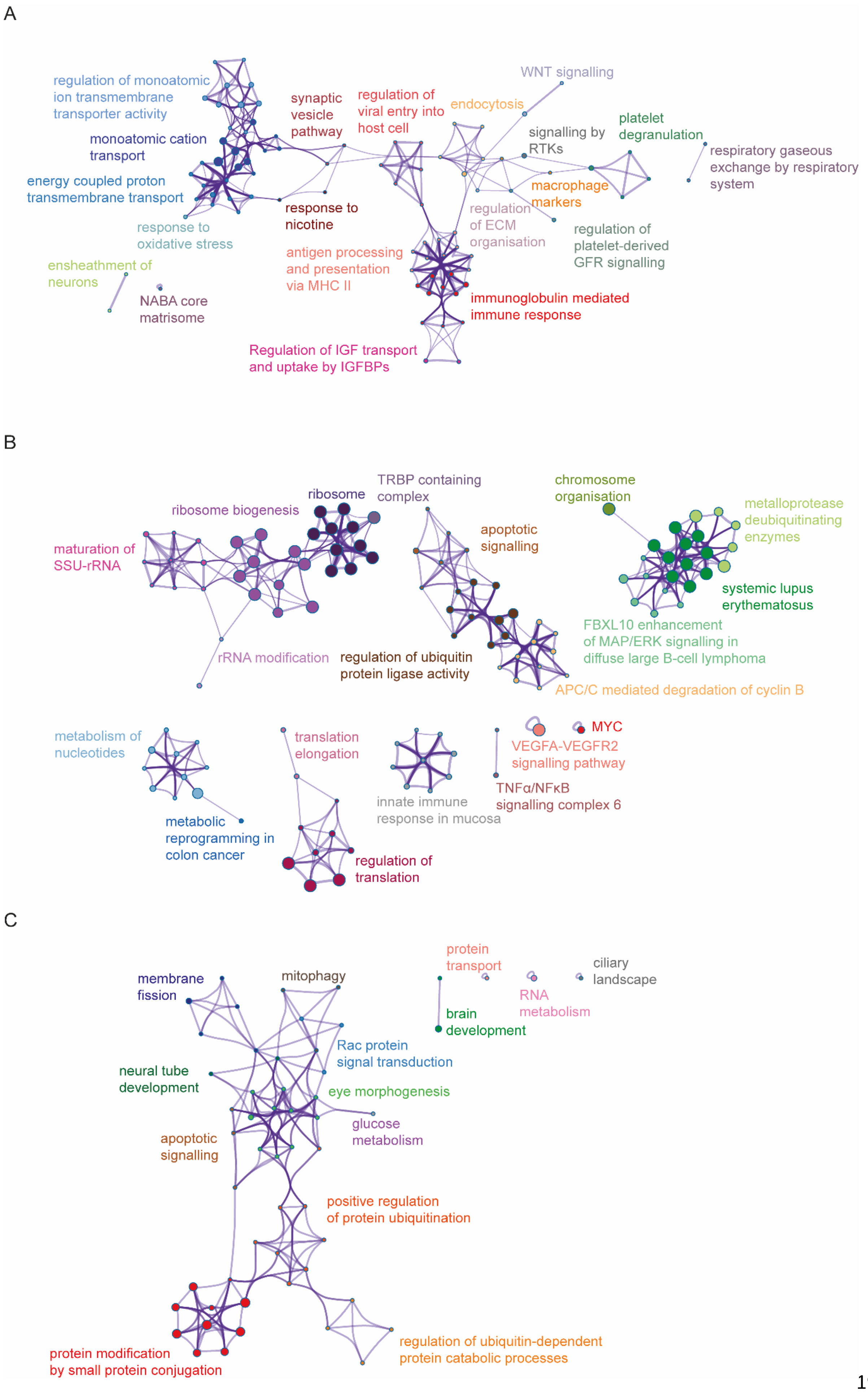
A
regulation of monoatomic ion transmembrane transporter activity
monoatomic cation transport
energy coupled proton transmembrane transport
response to oxidative stress
ensheathment of neurons
NABA core matrisome
synaptic vesicle pathway
response to nicotine
regulation of viral entry into host cell
antigen processing and presentation via MHC II
Regulation of IGF transport and uptake by IGFBPs
immunoglobulin mediated immune response
endocytosis
WNT signalling
signalling by RTKs
platelet degranulation
respiratory gaseous exchange by respiratory system
macrophage markers
regulation of ECM organisation
regulation of platelet-derived GFR signalling
B
maturation of SSU-rRNA
ribosome biogenesis
ribosome
TRBP containing complex
apoptotic signalling
chromosome organisation
metalloprotease deubiquitinating enzymes
systemic lupus erythematosus
FBXL10 enhancement of MAP/ERK signalling in diffuse large B-cell lymphoma
rRNA modification
regulation of ubiquitin protein ligase activity
APC/C mediated degradation of cyclin B
metabolism of nucleotides
metabolic reprogramming in colon cancer
translation elongation
regulation of translation
innate immune response in mucosa
VEGFA-VEGFR2 signalling pathway
MYC
TNFα/NFκB signalling complex 6
C
membrane fission
mitophagy
protein transport
RNA metabolism
ciliary landscape
brain development
Rac protein signal transduction
neural tube development
eye morphogenesis
glucose metabolism
apoptotic signalling
positive regulation of protein ubiquitination
protein modification by small protein conjugation
regulation of ubiquitin-dependent protein catabolic processes

Fig. S5, related to Fig. 2: Metascape DEG analysis of G3/G4 clusters from ICGC cohort

Networks showing clusters of significant pathways identified within the scope of DEG analyses employing Metascape. Each node represents one pathway with node size referring to the number of matching DEGs and node colour indicating its affiliation to a particular pathway cluster. Lines connect similar pathways (similarity score > 0.3 calculated by Metascape) with the thickness of a line illustrating the similarity score. DEG lists of A) I_G3/4.1, B) I_G3/4.2 and C) I_G3/4.3 comparing only G3/G4 clusters of the ICGC cohort have been used. For networks A) and B), only DEGs with a logFC≥1 were taken into account, in accordance with Additional file 1: Fig. S4. Network C) includes all DEGs of I_G3/4.3 with a logFC>0 due to the cluster's small number of DEGs.

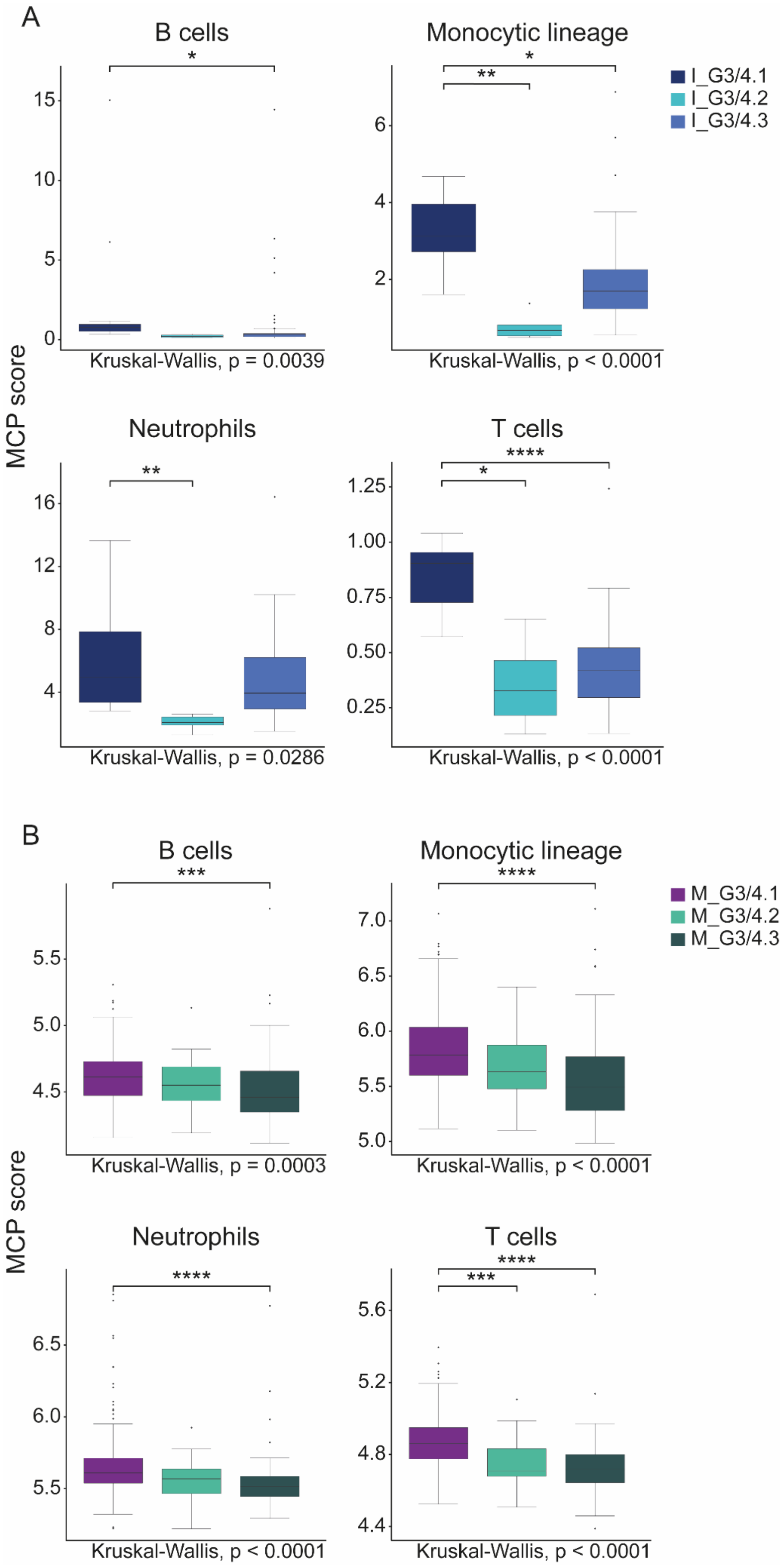
A
B cells
*
15
10
5
0
Kruskal-Wallis, p = 0.0039
Monocytic lineage
**
*
6
4
2
Kruskal-Wallis, p < 0.0001
I_G3/4.1
I_G3/4.2
I_G3/4.3
MCP score
Neutrophils
**
16
12
8
4
Kruskal-Wallis, p = 0.0286
T cells
****
*
1.25
1.00
0.75
0.50
0.25
Kruskal-Wallis, p < 0.0001
B
B cells
***
5.5
5.0
4.5
Kruskal-Wallis, p = 0.0003
Monocytic lineage
****
7.0
6.5
6.0
5.5
5.0
Kruskal-Wallis, p < 0.0001
M_G3/4.1
M_G3/4.2
M_G3/4.3
MCP score
Neutrophils
****
6.5
6.0
5.5
Kruskal-Wallis, p < 0.0001
T cells
****
***
5.6
5.2
4.8
4.4
Kruskal-Wallis, p < 0.0001

Fig. S6, related to Fig. 2: Computational quantification of cell populations across metabolic clusters

Boxplots showing abundance scores of particular cell types from the TME calculated with MCP-counter across G3/G4 clusters for A) ICGC and B) MAGIC cohort. Scores for B cells, the monocytic lineage, neutrophils and T cells are shown. Results of Kruskal-Wallis and Wilcoxon rank-sum tests are displayed for each cell type. P-values have been adjusted using Bonferroni correction, and $p<0.05$ was considered significant. * = adjusted p-value < 0.05; ** = adjusted p-value ≤ 0.01; *** = adjusted p-value ≤ 0.001; **** = adjusted p-value ≤ 0.0001

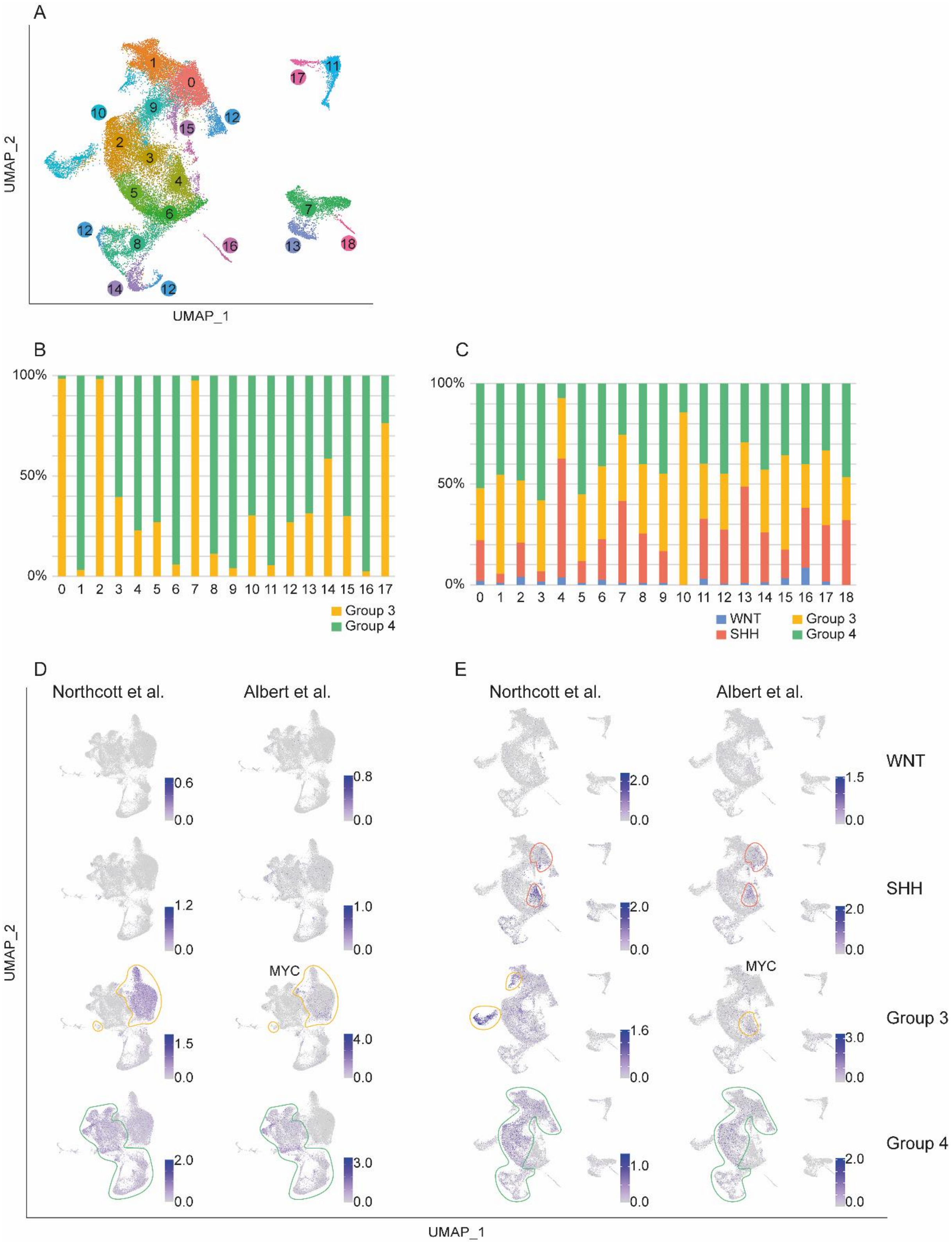

Fig. S7, related to Fig. 3: Annotation of single-cell RNA clusters concerning tumour group

A) Integrated clustering of human MB single-cell transcriptomes from Riemondy et al. is depicted in a two-dimensional UMAP plot. The fraction of cells belonging to G3/G4 samples for every cluster is shown for both scRNA-seq cohorts, with B) referring to MSMB and C) to RMB. D), E) Published RNA expression programs of MB tumour groups(16,17) have been plotted for D) MSMB and E) RMB cohorts. Results from this figure have been used for tumour group annotation in Fig. 3.

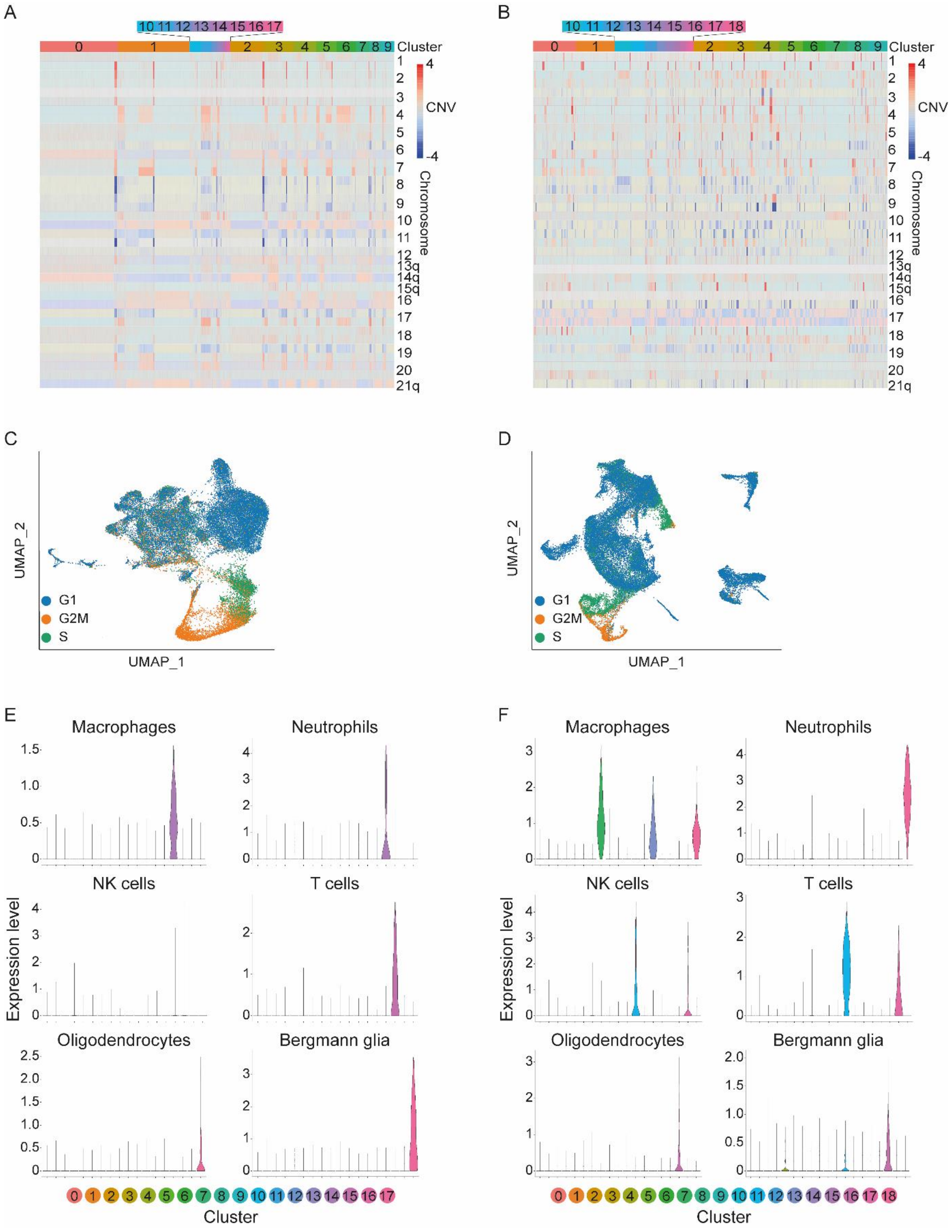

Fig. S8, related to Fig. 3: Annotation of single-cell RNA clusters concerning cell type

A) and B) show CNVs based on scRNA-seq expression data, which have been analysed to identify tumour cell clusters. C), D) Depiction of cell-cycle phases based on gene signatures. E), F) Violin plots illustrating the expression marker gene signatures for cells of the TME shown in Fig. 3. A list of genes for each signature can be found in Additional file 7: Table S6. Figure panels A), C) and E) refer to the MSMB dataset, while panels B), D) and F) show results for the dataset from Riemondy et al.

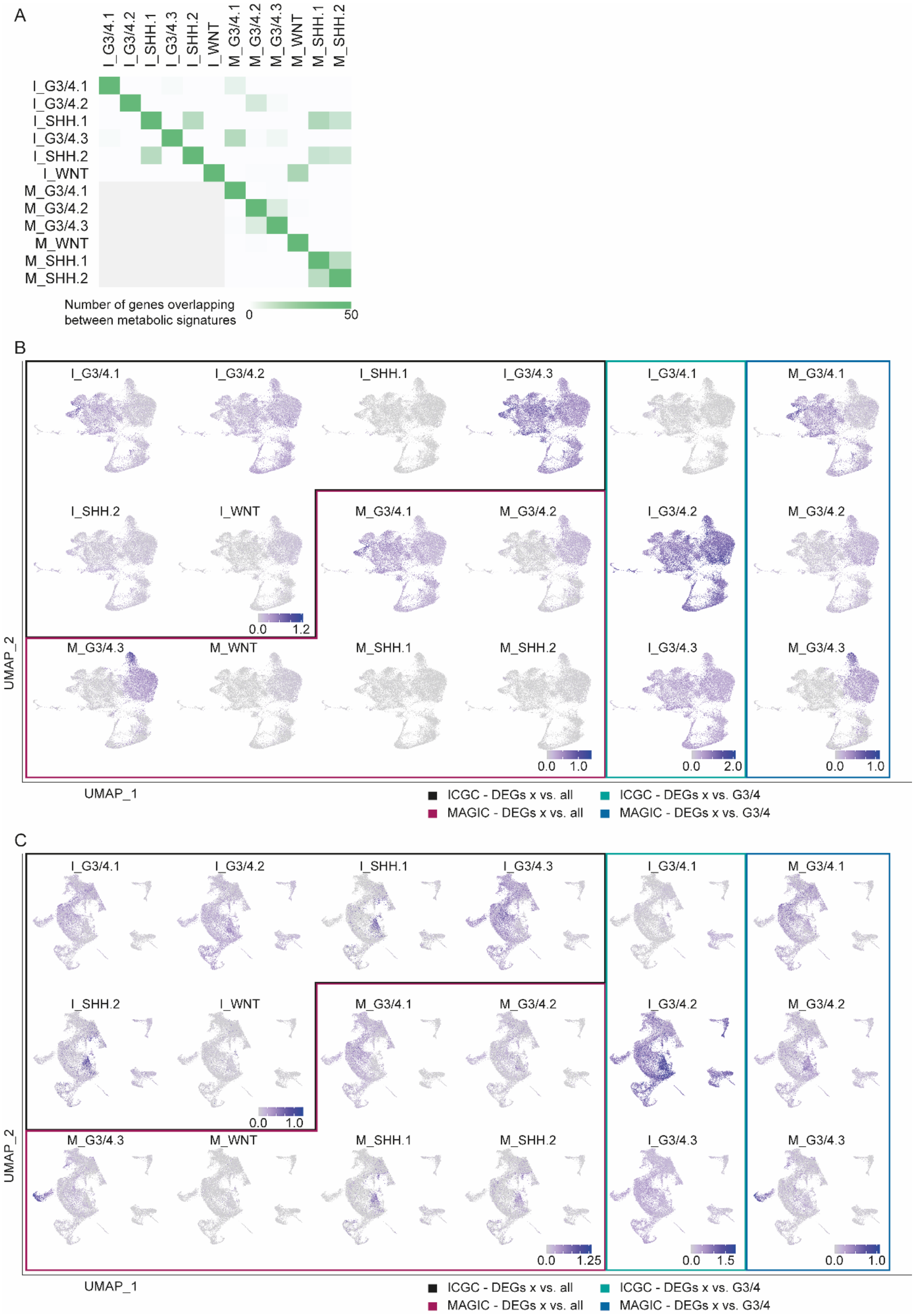
A
I_G3/4.1
I_G3/4.2
I_SHH.1
I_G3/4.3
I_SHH.2
I_WNT
M_G3/4.1
M_G3/4.2
M_G3/4.3
M_WNT
M_SHH.1
M_SHH.2
Number of genes overlapping between metabolic signatures
0
50
B
UMAP_2
UMAP_1
ICGC - DEGs x vs. all
MAGIC - DEGs x vs. all
ICGC - DEGs x vs. G3/4
MAGIC - DEGs x vs. G3/4
C

Fig. S9, related to Fig. 3: Expression patterns of metabolic MB clusters at single-cell RNA level

A) Comparison of metabolic gene signatures (top 50 DEGs) derived from bulk RNA analysis. B), C) UMAPs displaying metabolic gene signatures. B) illustrates the results for MSMB and C) for RMB. Colours indicate which set of DEGs has been used, as shown below. Gene expression values covered by the UMAP legend were aligned across each set of DEG lists.

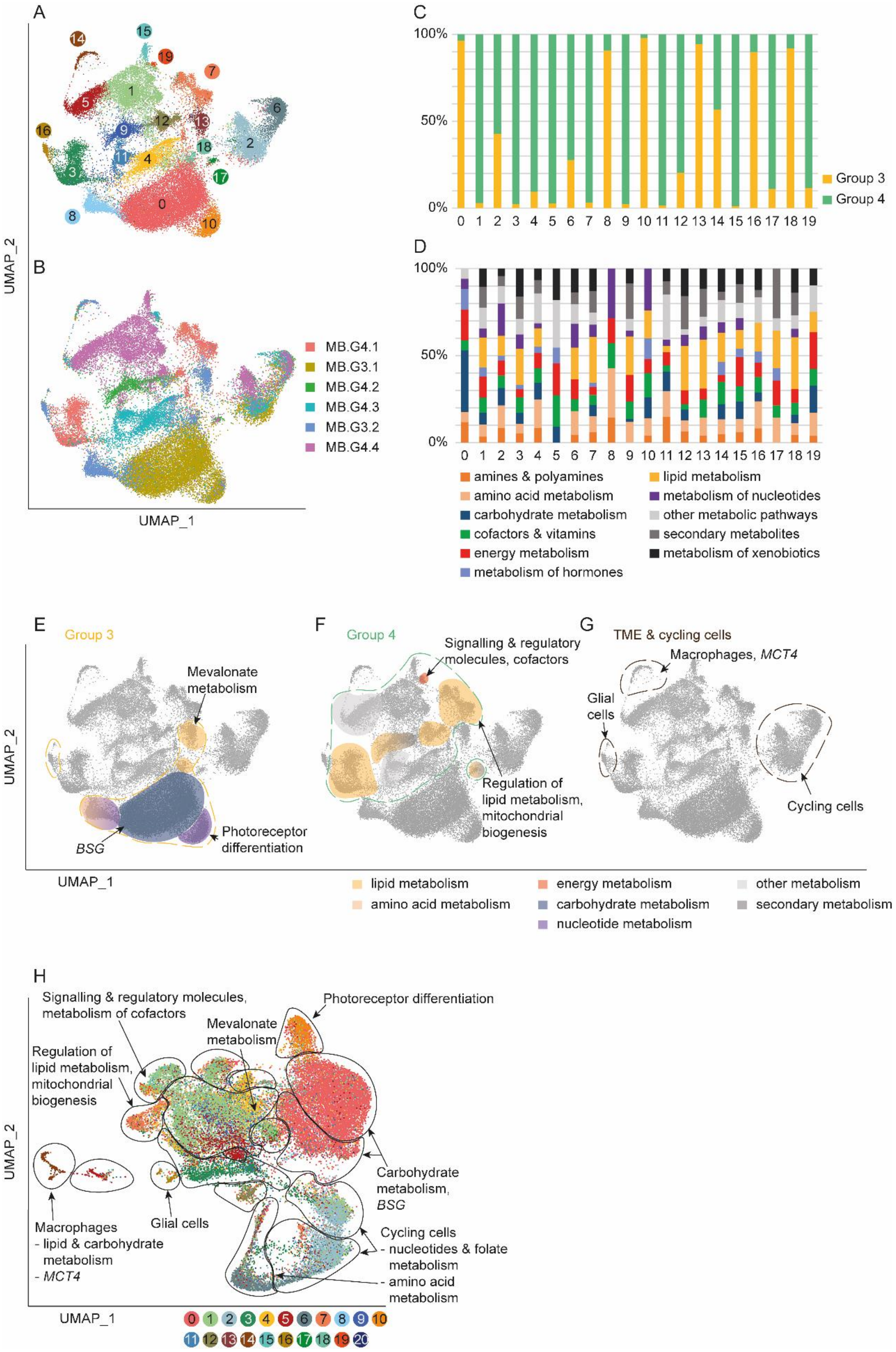

Fig. S10, related to Fig. 3: Analysis of metabolic UMAP clusters in the MSMB dataset

A) UMAP of MSMB dataset based on metabolic genes from ccmGDB. B) Cells cluster predominantly according to their tumour of origin. C) The cells per cluster belonging to each MB group are shown. D) Bar chart illustrating the fraction of analysed DEGs belonging to a specific metabolic category listed on IPA. E)-G) Characterisation of clusters composed of cells from E) G3 samples, F) G4 samples, and G) cells from the TME based on analyses of upregulated DEGs and of highly expressed unique genes (logFC≥1). H) UMAP based on whole single-cell transcriptomes with black lines referring to clusters, as shown in Fig. 3A. Colours refer to clustering based on metabolic genes depicted in panel A) of this figure. Annotation of cell types and metabolic characteristics as outlined in previous plots have been summarised.

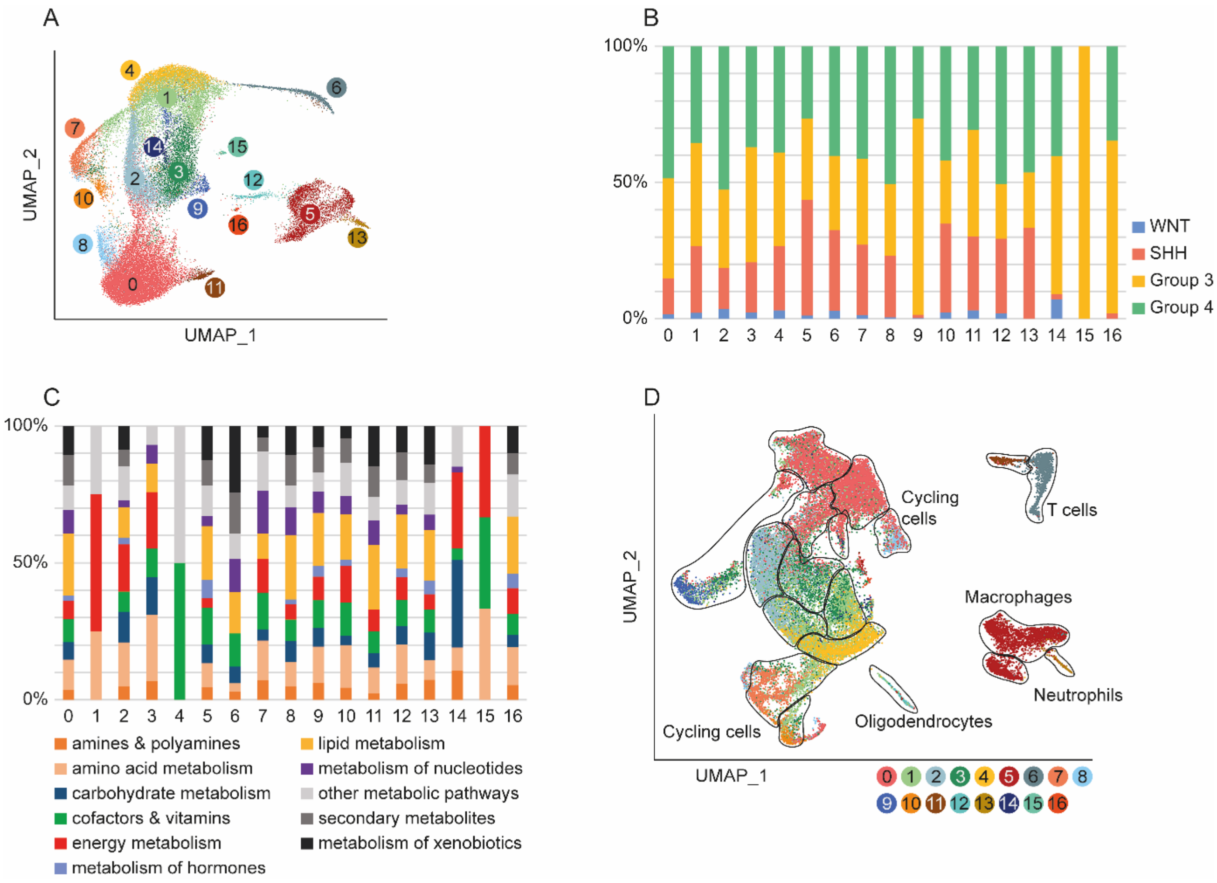

Fig. S11, related to Fig. 3: Metabolic UMAP clusters in the RMB dataset

Results are shown in line with Additional file 1: Fig. S10. A) Metabolic UMAP of RMB dataset. B) Bar chart showing the cells per cluster belonging to the each MB group. C) Plot depicting metabolic categories listed on IPA and the fraction of analysed DEGs matching the according categories. D) UMAP based on whole single-cell transcriptomes with black lines referring to clusters of the standard UMAP (Additional file 1: Fig. S7A) and colours indicating the clustering based on metabolic genes.

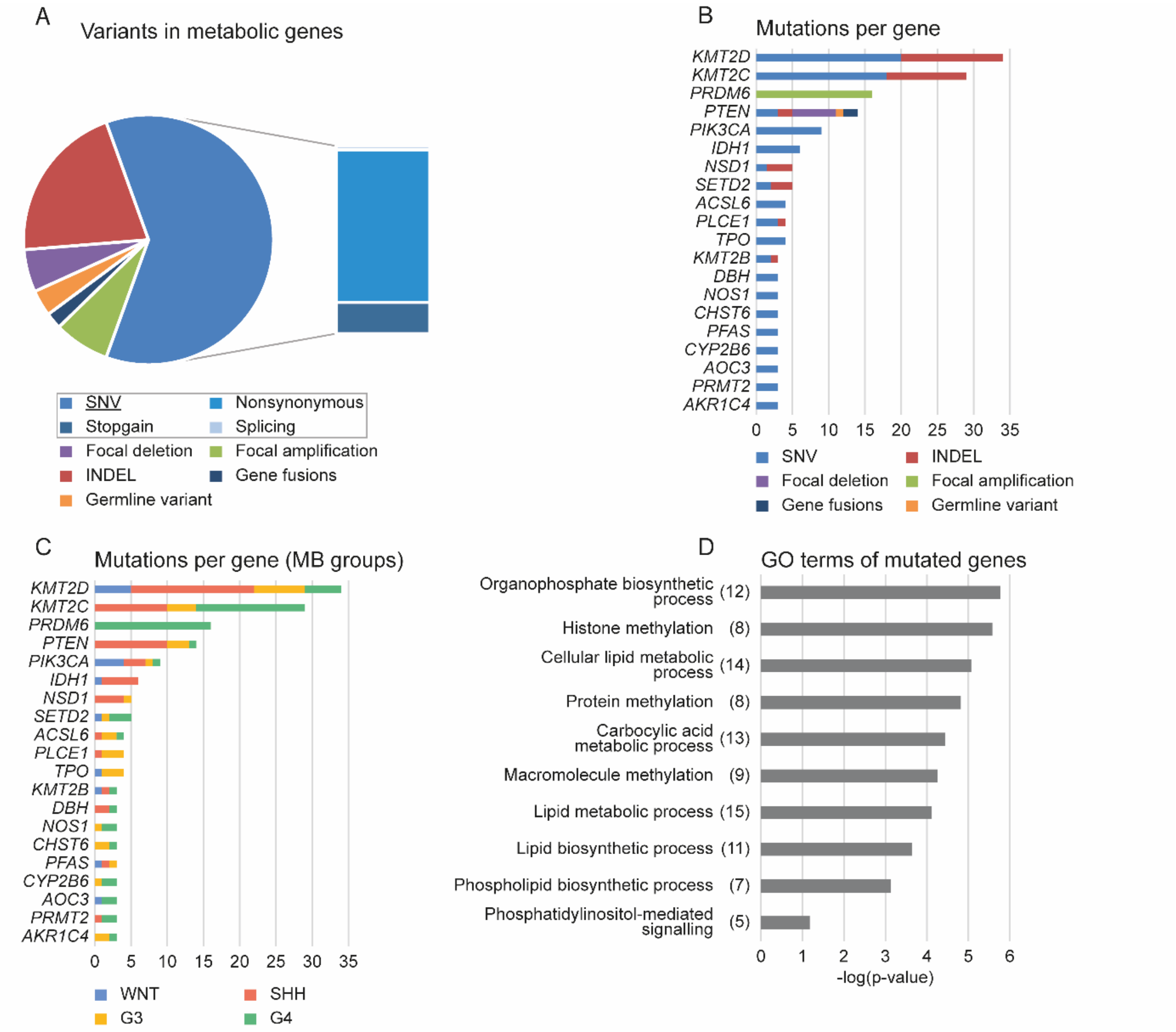

Fig. S12, related to Fig. 4: Validation of nDNA analysis utilising metabolic genes from Rosario et al.

Analysis of aberrations in metabolic genes was repeated using the second list of metabolic genes(8) in line with bulk RNA analysis. 236 mutations in 40 genes were detected. A) depicts the mutation type of all variants, including a further classification of SNVs as nonsynonymous, splicing and stopgain mutations. Details on B) mutation type and C) MB group affiliation of the top 20 mutated genes are illustrated. D) GO analysis of all mutated genes using ToppGene Suite revealed that lipid metabolism can also be found in this validation, although with a higher p-value. The number of

genes found for each GO term is shown in brackets. SNV = single nucleotide variant; INDEL = insertions and deletions

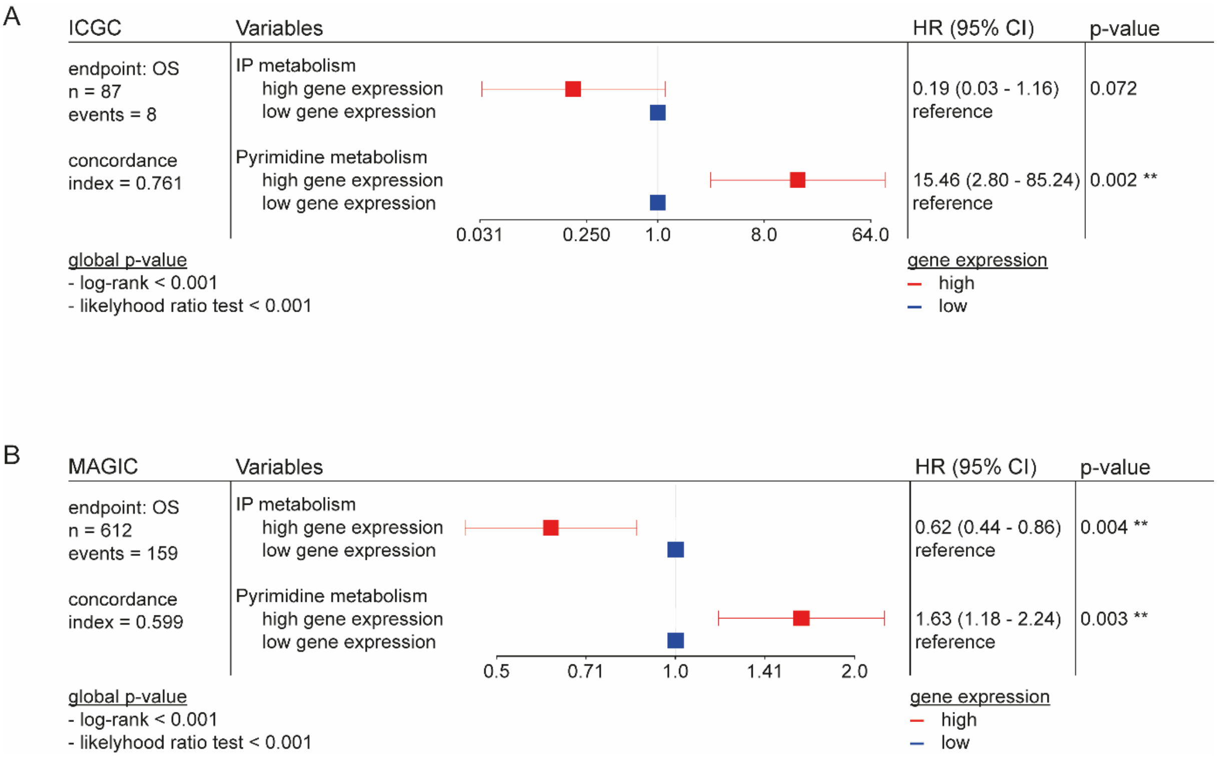

Fig. S13, related to Fig. 5: Multivariate Cox regression analysis

Forest plots depicting the results of multivariate cox regression analysis of A) ICGC cohort and B) MAGIC cohort are shown. Squares represent the according hazard ratios, and error bars refer to confidence intervals. Both cohorts were stratified for MB groups. For metabolic pathways, the group “low gene expression” by maxstat was taken as reference. HR = hazard ratio; CI = confidence interval; ** = p-value ≤ 0.01

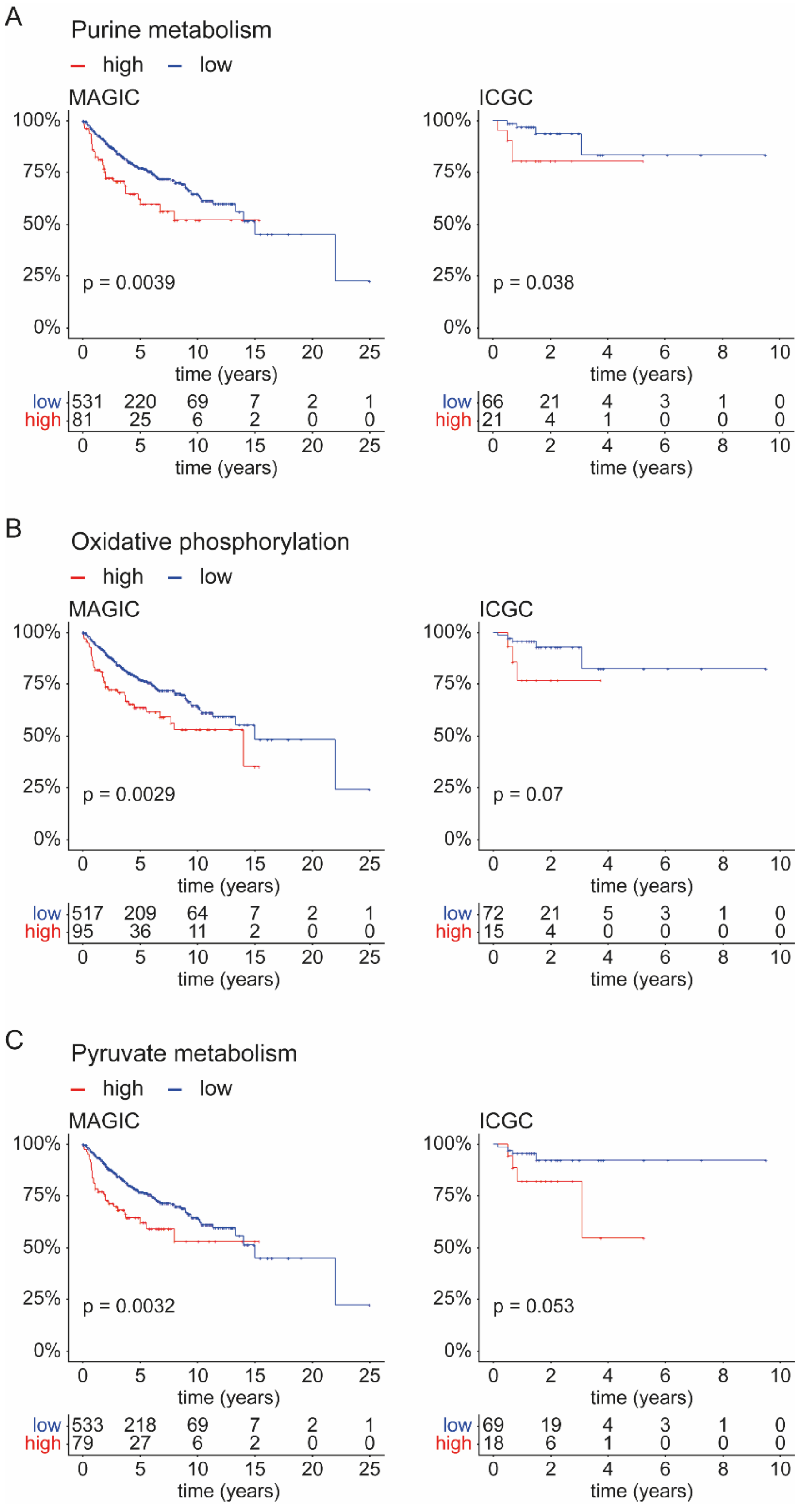

Fig. S14, related to Fig. 5: Prognostic relevance of energy metabolic pathways and purine metabolism

Kaplan-Meier curves show the overall survival of patients from MAGIC and ICGC cohorts. Patients have been divided into two groups depending on RNA expression levels of genes involved in the metabolism of A) purines, B) oxidative phosphorylation, and C) pyruvate metabolism. Log-rank test was used to calculate p-values, and $p<0.05$ was considered significant. Grouping patients was performed by maximally ranked statistics but was not significant for the pathways shown here.

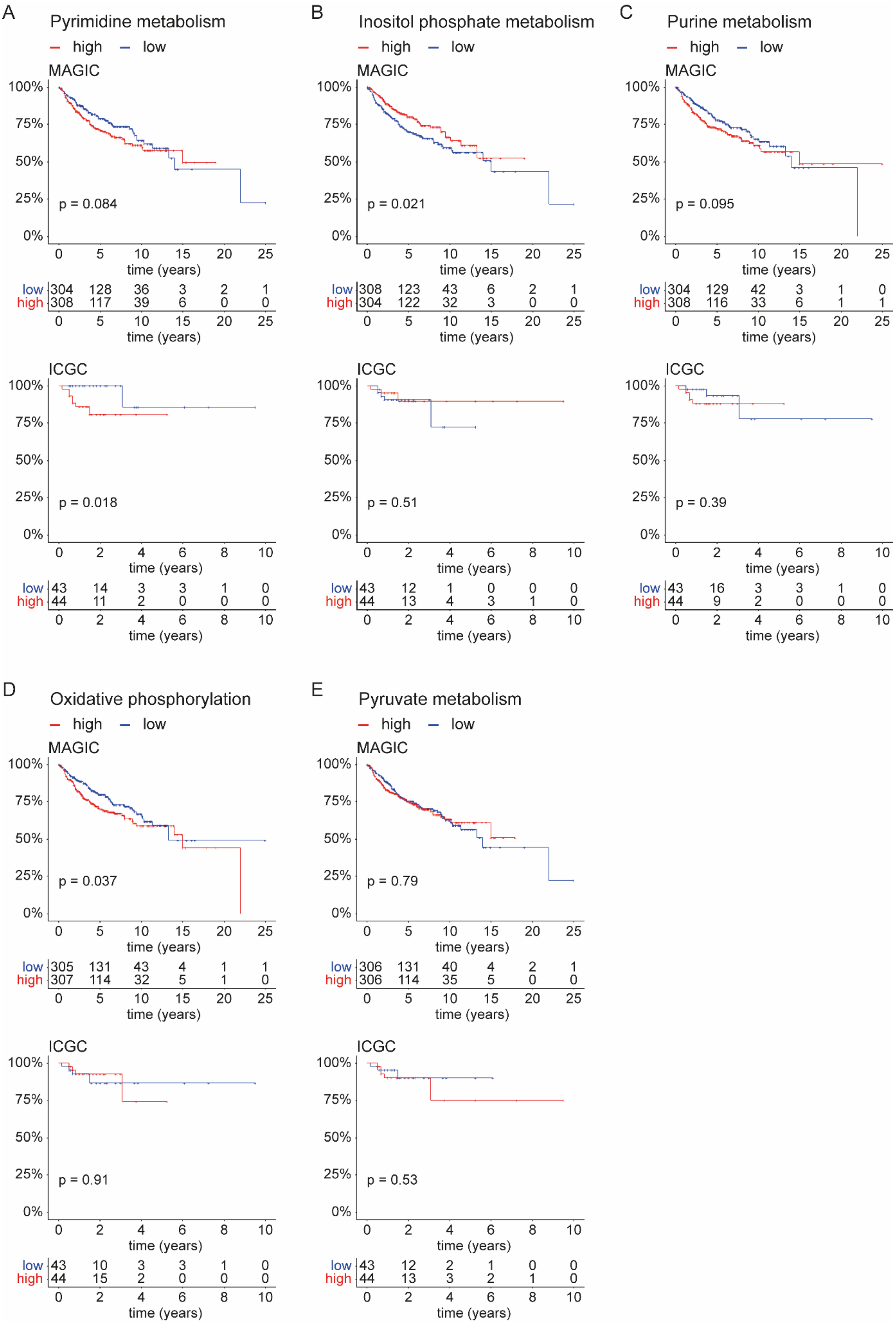
A
Pyrimidine metabolism
high low
MAGIC
100% 75% 50% 25% 0%
p = 0.084
0 5 10 15 20 25
time (years)
low 304 128 36 3 2 1
high 308 117 39 6 0 0
ICGC
p = 0.018
0 2 4 6 8 10
low 43 14 3 3 1 0
high 44 11 2 0 0 0
B
Inositol phosphate metabolism
high low
MAGIC
p = 0.021
low 308 123 43 6 2 1
high 304 122 32 3 0 0
ICGC
p = 0.51
low 43 12 1 0 0 0
high 44 13 4 3 1 0
C
Purine metabolism
high low
MAGIC
p = 0.095
low 304 129 42 3 1 0
high 308 116 33 6 1 1
ICGC
p = 0.39
low 43 16 3 3 1 0
high 44 9 2 0 0 0
D
Oxidative phosphorylation
high low
MAGIC
p = 0.037
low 305 131 43 4 1 1
high 307 114 32 5 1 0
ICGC
p = 0.91
low 43 10 3 3 1 0
high 44 15 2 0 0 0
E
Pyruvate metabolism
high low
MAGIC
p = 0.79
low 306 131 40 4 2 1
high 306 114 35 5 0 0
ICGC
p = 0.53
low 43 12 2 1 0 0
high 44 13 3 2 1 0

Fig. S15, related to Fig. 5: Grouping utilising the median gene expression as cut-off value yields fewer significant results

Overall survival of patients from MAGIC and ICGC cohorts similar to Kaplan-Meier curves shown before (Fig. 5; Additional file 1: Fig. S14) for A) pyrimidine, B) IP, C) purine metabolism, D) oxidative phosphorylation, and E) pyruvate metabolism. Division of patients into two groups depending on RNA expression levels of genes involved in previously analysed pathways has been done using the median gene expression level as cut-off value. Significance was calculated as mentioned above.

3. Supplementary References

1. R Core Team. R: A Language and Environment for Statistical Computing. R Foundation for Statistical Computing, Vienna, Austria; 2021. https://www.R-project.org/
2. Kolde R. pheatmap: Pretty Heatmaps. R package version 1.0.12; 2019. https://CRAN.R-project.org/package=pheatmap
3. Kim P, Cheng F, Zhao J, Zhao Z. ccmGDB: a database for cancer cell metabolism genes. Nucleic Acids Res. 2016;44(D1):D959–68.
4. Maechler M, Rousseeuw P, Struyf A, Hubert M, Hornik K, Studer M, et al. cluster: Cluster Analysis Basics and Extensions. 2021. https://CRAN.R-project.org/package=cluster
5. Love MI, Huber W, Anders S. Moderated estimation of fold change and dispersion for RNA-seq data with DESeq2. Genome Biol. 2014;15(12):550.

6. Kassambara A, Kosinski M, Biecek P. survminer: Drawing Survival Curves using “ggplot2”. R package version 0.4.9; 2021. https://CRAN.R-project.org/package=survminer
7. Ritchie ME, Phipson B, Wu D, Hu Y, Law CW, Shi W, et al. limma powers differential expression analyses for RNA-sequencing and microarray studies. Nucleic Acids Res. 2015;43(7):e47.
8. Rosario SR, Long MD, Affronti HC, Rowsam AM, Eng KH, Smiraglia DJ. Pan-cancer analysis of transcriptional metabolic dysregulation using The Cancer Genome Atlas. Nat Commun. 2018;9(1):5330.
9. Zhou Y, Zhou B, Pache L, Chang M, Khodabakhshi AH, Tanaseichuk O, et al. Metascape provides a biologist-oriented resource for the analysis of systems-level datasets. Nat Commun. 2019;10(1):1523.
10. R Core Team. R: A Language and Environment for Statistical Computing. R Foundation for Statistical Computing, Vienna, Austria; 2022. https://www.R-project.org/
11. Becht E, Giraldo NA, Lacroix L, Buttard B, Elarouci N, Petitprez F, et al. Estimating the population abundance of tissue-infiltrating immune and stromal cell populations using gene expression. Genome Biol. 2016;17(1):218.
12. Allaire JJ, Gandrud C, Russell K, Yetman CJ. networkD3: D3 JavaScript Network Graphs from R. R package version 0.4; 2017. https://CRAN.R-project.org/package=networkD3
13. Riemondy KA, Venkataraman S, Willard N, Nellan A, Sanford B, Griesinger AM, et al. Neoplastic and immune single-cell transcriptomics define subgroup-specific intra-tumoral heterogeneity of childhood medulloblastoma. Neuro Oncol. 2022;24(2):273–86.

14. Zheng GXY, Terry JM, Belgrader P, Ryvkin P, Bent ZW, Wilson R, et al. Massively parallel digital transcriptional profiling of single cells. Nat Commun. 2017;8:14049.
15. Hao Y, Hao S, Andersen-Nissen E, Mauck WM 3rd, Zheng S, Butler A, et al. Integrated analysis of multimodal single-cell data. Cell. 2021;184(13):3573-3587.e29.
16. Northcott PA, Shih DJH, Remke M, Cho YJ, Kool M, Hawkins C, et al. Rapid, reliable, and reproducible molecular sub-grouping of clinical medulloblastoma samples. Acta Neuropathol. 2012;123(4):615–26.
17. Albert TK, Interlandi M, Sill M, Graf M, Moreno N, Menck K, et al. An extracellular vesicle-related gene expression signature identifies high-risk patients in medulloblastoma. Neuro Oncol. 2021;23(4):586–98.
18. Ocasio J, Babcock B, Malawsky D, Weir SJ, Loo L, Simon JM, et al. scRNA-seq in medulloblastoma shows cellular heterogeneity and lineage expansion support resistance to SHH inhibitor therapy. Nat Commun. 2019;10(1):5829.
19. Vladoiu MC, El-Hamamy I, Donovan LK, Farooq H, Holgado BL, Sundaravadanam Y, et al. Childhood cerebellar tumours mirror conserved fetal transcriptional programs. Nature. 2019;572(7767):67–73.
20. Carter RA, Bihannic L, Rosencrance C, Hadley JL, Tong Y, Phoenix TN, et al. A Single-Cell Transcriptional Atlas of the Developing Murine Cerebellum. Curr Biol. 2018;28(18):2910-2920.e2.
21. Zeisel A, Hochgerner H, Lönnerberg P, Johnsson A, Memic F, van der Zwan J, et al. Molecular Architecture of the Mouse Nervous System. Cell. 2018;174(4):999-1014.e22.

22. Melcher V, Graf M, Interlandi M, Moreno N, de Faria FW, Kim SN, et al. Macrophage-tumor cell interaction promotes ATRT progression and chemoresistance. Acta Neuropathol. 2020;139(5):913–36.
23. Margol AS, Robison NJ, Gnanachandran J, Hung LT, Kennedy RJ, Vali M, et al. Tumor-associated macrophages in SHH subgroup of medulloblastomas. Clin Cancer Res. 2015;21(6):1457–65.
24. Tirosh I, Izar B, Prakadan SM, Wadsworth MH 2nd, Treacy D, Trombetta JJ, et al. Dissecting the multicellular ecosystem of metastatic melanoma by single-cell RNA-seq. Science. 2016;352(6282):189–96.
25. Müller S, Cho A, Liu SJ, Lim DA, Diaz A. CONICS integrates scRNA-seq with DNA sequencing to map gene expression to tumor sub-clones. Bioinformatics. 2018;34(18):3217–9.
26. Rue-Albrecht K, Marini F, Soneson C, Lun ATL. iSEE: Interactive SummarizedExperiment Explorer. F1000Res. 2018;7:741.
27. Northcott PA, Buchhalter I, Morrissy AS, Hovestadt V, Weischenfeldt J, Ehrenberger T, et al. The whole-genome landscape of medulloblastoma subtypes. Nature. 2017;547(7663):311-7.
28. Hothorn T. maxstat: Maximally Selected Rank Statistics. R package version 0.7-25; 2017. https://CRAN.R-project.org/package=maxstat
29. Lausen B, Hothorn T, Bretz F, Schumacher M. Assessment of Optimal Selected Prognostic Factors. Biom J. 2004;46(3):364–74.
30. Therneau TM. A Package for Survival Analysis in R. R package version 3.2-13; 2021. https://CRAN.R-project.org/package=survival
31. Gordon M, Lumley T. forestplot: Advanced Forest Plot Using “grid” Graphics. R package version 3.1.0; 2022. https://CRAN.R-project.org/package=forestplot

32. Sharma T, Schwalbe EC, Williamson D, Sill M, Hovestadt V, Mynarek M, et al. Second-generation molecular subgrouping of medulloblastoma: an international meta-analysis of Group 3 and Group 4 subtypes. Acta Neuropathol. 2019;138(2):309–26.
33. Cavalli FMG, Remke M, Rampasek L, Peacock J, Shih DJH, Luu B, et al. Intertumoral Heterogeneity within Medulloblastoma Subgroups. Cancer Cell. 2017;31(6):737-754.e6.
